# Recent advances in extracellular vesicle engineering and its applications to regenerative medicine

**DOI:** 10.1186/s40824-023-00468-6

**Published:** 2023-12-11

**Authors:** Won-Kyu Rhim, Jun Yong Kim, Seung Yeon Lee, Seung-Gyu Cha, Jeong Min Park, Hyeon Jeong Park, Chun Gwon Park, Dong Keun Han

**Affiliations:** 1 Department of Biomedical Science, CHA University, 335 Pangyo-ro Bundang-gu, Seongnam-si, Gyeonggi-do 13488 Republic of Korea; 2 Department of Biomedical Engineering, 2066 Seobu-ro Jangan-gu, Suwon-si, Gyeonggi-do 16419 Republic of Korea; 3 Intelligent Precision of Healthcare Convergence, SKKU Institute for Convergence, Sungkyunkwan University (SKKU), 2066 Seobu-ro Jangan-gu, Suwon-si, Gyeonggi-do 16419 Republic of Korea

**Keywords:** Extracellular vesicles (EVs), Gene editing, Endogenous engineering, Exogenous engineering, Hybridization, Regenerative medicine

## Abstract

Extracellular vesicles (EVs) are nanosized particles that are released from cells and reflect the characteristics of the mother cell. Recently, the EVs have been used in several types of studies across many different fields. In the field of EV research, multiple cell culture and EV isolation techniques have been highlighted in importance. Various strategies, including exclusive component culture media, three-dimensional (3D) cultures, and hypoxic conditions, have been proposed for the cell culture to control function of the EVs. Ultracentrifugation, ultrafiltration, precipitation, and tangential flow filtration (TFF) have been utilized for EV isolation. Although isolated EVs have their own functionalities, several researchers are trying to functionalize EVs by applying various engineering approaches. Gene editing, exogenous, endogenous, and hybridization methods are the four well-known types of EV functionalization strategies. EV engineered through these processes has been applied in the field of regenerative medicine, including kidney diseases, osteoarthritis, rheumatoid arthritis, nervous system-related diseases, and others. In this review, it was focused on engineering approaches for EV functionalization and their applications in regenerative medicine.

**Graphical Abstract:**

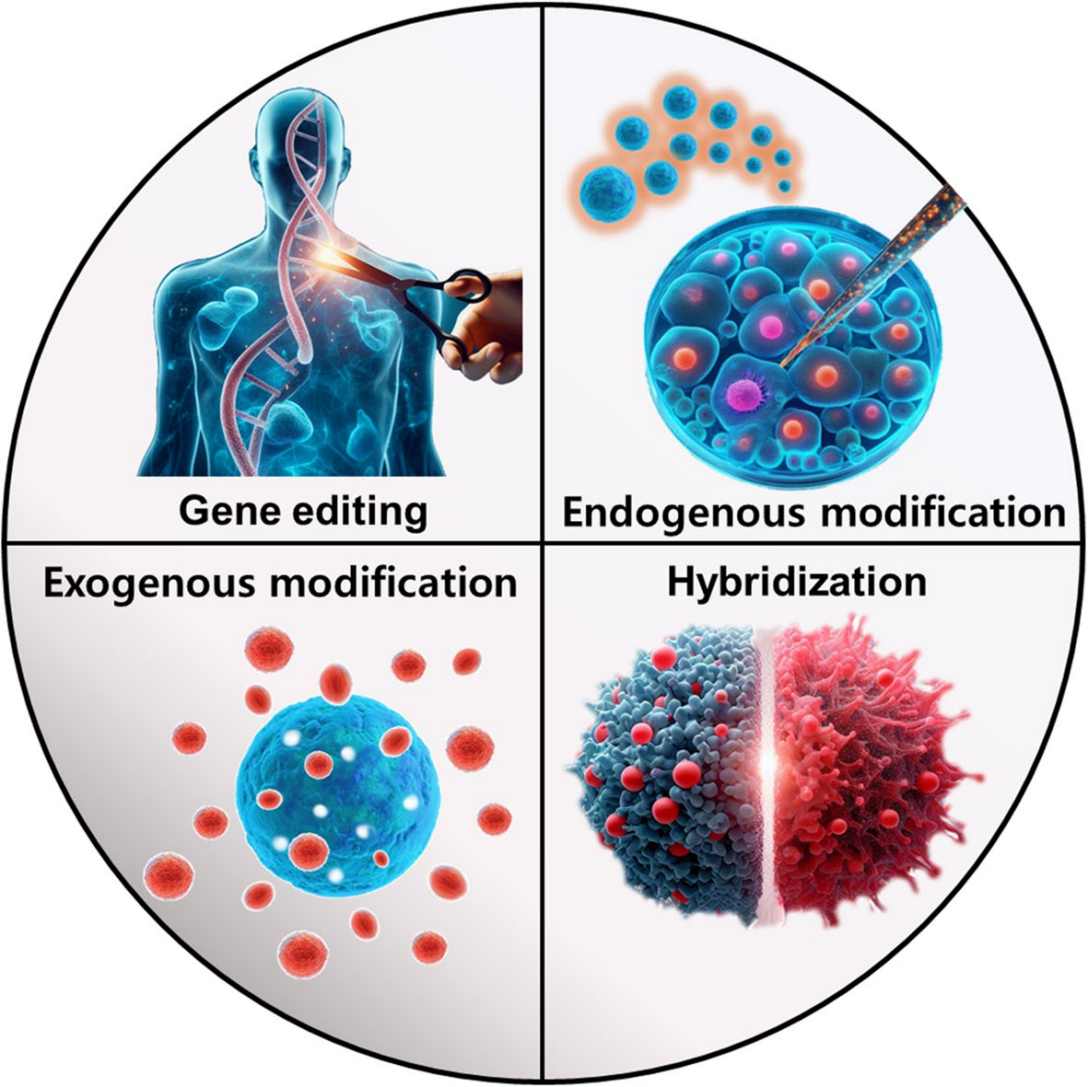

## Background

**Fig. 1:**
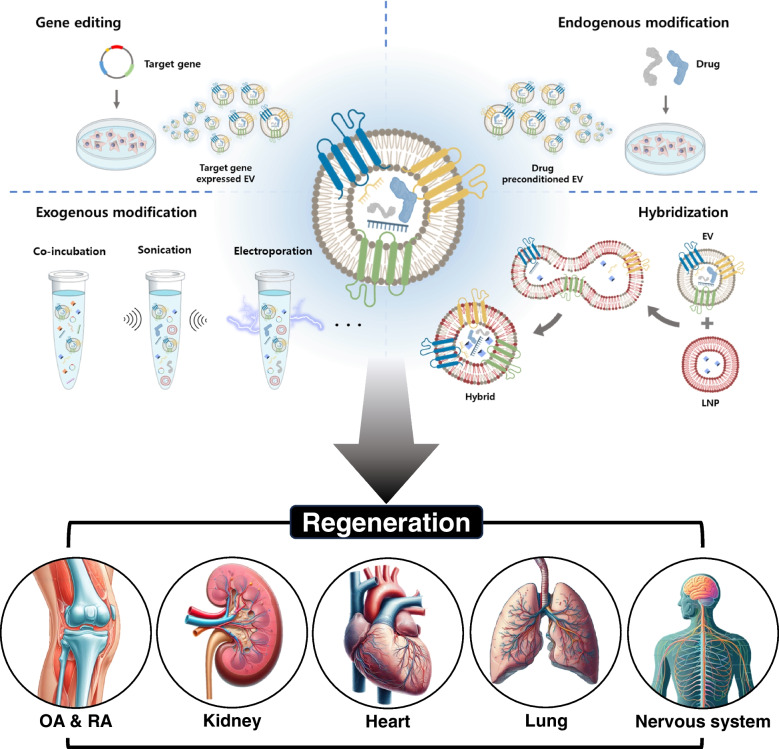
Schematic illustrations of EV engineering methods and various applications for regenerative medicine

In recent years, there has been a surge in utilizing the potential of extracellular vesicles (EVs) in a wide range of research fields [1–4]. The EVs reflect the characteristics of their parent cells and are typically recognized as particles of a size from 30 to 150 nm [5, 6]. In order to optimize the production and isolation of EVs, various methods for cell culture and EV isolation have been proposed. The controlling conditions for cell culturing contain media components, a three-dimensional (3D) culture system, and hypoxic conditions. It is known that the fetal bovine serum (FBS), commonly used to support cell growth during the cell culture process, contains its own EVs. There have been reports suggesting that FBS-derived EVs can potentially act as animal originated contaminants for EVs from targeted cells [7]. To address these issues, some efforts have been made to use Xeno-free media (XFM) that contain supplement formulations to allow cell growth. Additionally, the formation of 3D spheroids as an approach to isolate an increased quantity and improved functionality of EVs has been reported [8, 9]. A recent study has presented that hypoxic conditions enhance both the quantity and functionality of EVs released from cells [10]. There are various methods for EV isolations, such as ultracentrifugation (UC), ultrafiltration (UF), precipitation, and tangential flow filtration (TFF) [11–13]. Despite the high purity of the traditional separation method, UC, TFF based separation has been widely utilized recently to overcome the problem of UC, such as extremely low production yield and long-lasting process time. Beyond these methods, various other EV isolation methods are continually being developed and applied across diverse fields, and research is underway to equip them with additional functions beyond their inherent effects. These engineering strategies can be broadly categorized into four main methods such as “gene editing,” “endogenous modification,” “exogenous modification,” and “hybridization” (Fig. 1). In gene editing approaches, transfection is predominantly utilized to engineer systems within target cells, enabling the production of desired proteins or the encapsulation of specific substances into EVs. The endogenous approach includes the treatment of cells with substances capable of modifying cell characteristics, with the goal of obtaining EVs derived from these altered cells. Generally, the treatment of TNF-α or IFN-γ to mesenchymal stem cells (MSCs) to obtain EVs with improved functionality is a process also known as “cell priming.” [14] In addition, the preconditioning involving both inflammatory factors and differentiation-inducing agents like TGFβ3, as well as antioxidants such as melatonin (Mel), resveratrol (Res), and curcumin (Cur), is applied for production of functionalized EVs [15–18]. The exogenous approach, a method for engineering isolated EVs, is characterized by the application of external forces for engineering except for simple incubation methods. These methods include simple incubation, electroporation, sonication, extrusion, freeze and thaw, etc.. In a simple incubation, substances intended for loading into EVs are dissolved in an EV-containing solution, allowing them to diffuse into the interior of the EVs. Electroporation is a technique employed in cell transformation or transfection in which external electrical stimulation is applied to induce the formation of small pores in the lipid bilayer of EVs, allowing the entry of external substances. Sonication is a method that utilizes ultrasound to weaken lipid bilayer interactions in EVs, facilitating the entry of external materials. The extrusion method involves the continuous passage of EVs through a membrane with uniform pore size to homogenize their size and enable the entry of external molecules into their interior. And, the freeze and thaw method involves repeatedly freezing EVs in a low-temperature environment, such as liquid nitrogen, and then thawing them at room temperature or elevated temperatures. This process disrupts the lipid bilayer interactions of EVs, allowing the entry of external substances. As previously mentioned, it can be observed that most exogenous methods aim to loosen the lipid bilayer interactions of EVs, thus facilitating the loading of intended substances. Lastly, hybridization methods have been reported to induce the formation of combined particles with dual characteristics by facilitating interactions between EVs and particles composed of different lipids. Generally, researchers have frequently reported the use of liposomes as particles composed of different lipids in these methods. Additionally, the researches about hybridization between EVs and cell-derived nanovesicles (NV) or ghosts have been reported [19, 20]. These are utilized to improve functionalities of EVs and are valuable approaches not only for loading the intended substances into their interior but also for imparting functionality on the surface of the particles. The process of hybridization is facilitated by polyethylene glycol (PEG) incubation, extrusion after sonication, freeze and thaw, and others. It often capitalizes on the property of promoting lipid-lipid interactions via PEG or utilizes processes like exogenous approaches to loosen lipid-binding interactions. Although EVs engineered through the various methods mentioned above have been utilized across diverse fields, in this review, our focus is on their applications on regenerative medicine, such as kidney regeneration, osteoarthritis (OA), rheumatoid arthritis (RA), and nerve system regeneration [21–24].

## Preparations for EV isolation

### Culture conditions for isolation of EV

#### Culture media

**Fig. 2:**
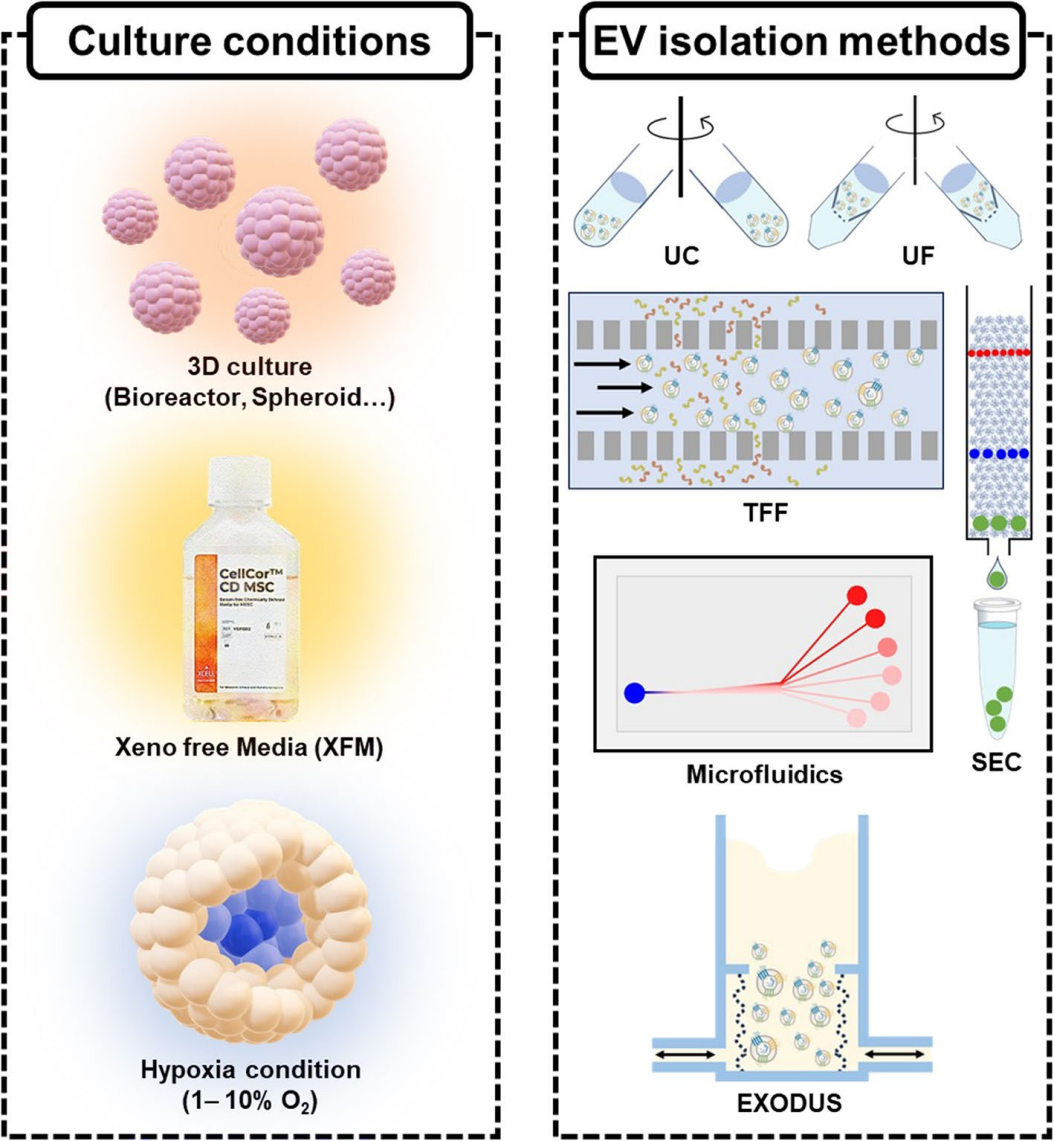
Common strategies of **A** cell culturing and **B** isolation methods for extracellular vesicles

The culture conditions of the cells significantly influence the characteristics of the EVs subsequently isolated. Researchers harness various culture conditions, including starvation, serum-free FBS, XFM, three-dimensional (3D) culture, hypoxia conditions, etc. (Fig. 2A). Starvation conditions expose cells to media without FBS for a specified duration after the cells have reached a certain confluency, leading to the isolation of cell derived EVs. Although many researchers have adopted this method, it can induce inflammation in cells, potentially resulting in the presence of inflammatory factors within isolated EVs [25]. In order to reduce cellular damage, researchers often utilize serum-free FBS [26]. Serum-free FBS is FBS that has been depleted of its inherent EVs using various EV isolation methods. However, many studies have revealed that serum-free FBS still contains a significant number of EVs derived from FBS, which can potentially affect the characteristics of EVs originating from cells [27]. Therefore, a novel approach designed to address the disadvantages of starvation and serum-free FBS conditions is the use of XFM. The XFM is a cell culture media that minimizes or completely excludes animal-derived components. Furthermore, various commercially available XFM products have been reported in the results of several researchers (Table 1).

**Table 1:** Commercially available Xeno-free media (XFM)

No.	Product name	Company	Country	Ref.
1	MSC NutriStem® XF	Sartorius	Germany	[28]
2	CellCor™ CD MSC	Xcell Therapeutics	Korea	[25]
3	KBM ADSC4	KOHJIN BIO	Japan	[29]
4	MSC Xeno-Free Culture Medium	Cellartis	Japan	[30]
5	StemXVivo Xeno-Free Human MSC Expansion Media	R&D Systems™	USA	[31]
6	RoosterNourish™-MSC-XF	RoosterBio	USA	[32]
7	StemPro MSC SFM XenoFree	Gibco	USA	[33]

Although there may be variations in XFM products depending on the manufacturer, it can help prevent the induction of inflammation, as seen in the starvation method, or unwanted EV mixing, as observed in the serum-free FBS method. In recent studies, comparative experiments were conducted on several XFMs showing differences in the quantity, purity, and marker expression levels of EVs originating from cells depending on the type of media [29]. Moreover, XFM conditions reportedly result in a greater yield of EVs and exhibit enhanced regenerative functionalities than the starvation method [25].

#### Physical stimulations for cell culture

The environmental factors influencing EVs are not limited solely to the media conditions among the culture conditions. Research has revealed various results on the isolation and characterization of EVs through 3D culture methods. The methods of 3D culture include techniques such as bioreactors, 3D hollow fiber bioreactors, and spheroid cultures. Most of these results generally point out that the introduction of 3D culture leads to an increased EV yield per cell [34, 35]. In addition, the reports indicate that EVs derived from 3D culture show superior functional characteristics compared to those derived from 2D culture [8]. This functional enhancement is believed to be due to the similarity between the EVs derived from the 3D culture and those originating in vivo [36]. Hypoxia conditions, electric stimulation, and high-frequency acoustic stimulation are factors that can also influence the characteristics of EVs. The Notch pathway is impacted when MSCs are exposed to hypoxia conditions, and the angiogenic effect could be increased by the upregulated Jagged1 in EVs. Furthermore, the increased expression of HIF-α under hypoxia conditions can increase the secretion of EVs. The reports suggest that the application of electric stimulation may lead to increased EV secretion by both B16F1 and 3 T3 Swiss Albino cells [37]. Lastly, the exposure of cells to high-frequency acoustic waves can lead to an increase in EV secretion through the activation of the calcium-dependent ALIX-mediated pathway [38]. The results of various cell culture conditions demonstrate that they are essential factors influencing the characteristics of EVs.

### The isolation methods of EV

There has been extensive research into diverse methods for isolating EVs. Despite the existence of various methods, in this review, we introduce several representative isolation techniques, including UC, UF, tangential flow filtration (TFF), microfluidics, PEG precipitation, size exclusion chromatography (SEC), and immunoaffinity (Fig. 2B). The most widely utilized method is UC which uses strong centrifugal forces to separate EVs. This method is easily accessible but has the disadvantage of yielding a small amount of separated EVs and requiring a significant amount of separation time. The other method that uses centrifugal force is UF, which involves passing the sample through a cutoff filter at an appropriate speed centrifugation that effectively filters EVs based on their size. However, this method leads to filter cake formation and sample aggregation when a constant centrifugal force is applied in the filter direction. In contrast, the TFF method utilizes a cutoff filter like UF but is designed to allow waste to flow in a direction orthogonal to the sample flow, which prevents filter cake formation, which is its advantage. Moreover, when used in conjunction with 3D culture, there have been reports of the ability to isolate a greater quantity of EVs compared to the 2D-UC, 2D-TFF, and 3D-UC methods [39]. Cutoff filters commonly utilized in the TFF method include those with cutoff size of 300 kDa and 500 kDa. Research on the use of a 500 kDa cutoff filter allows for the isolation of relatively high-purity EVs [26]. In addition, scaling up the size of the filter for separation enables the processing of large sample volumes, which is advantageous for industrial applications. In the separation method using flow dynamics, microfluidics is also employed, which is a method that leverages particle behavior based on fluid flow, and various designs of devices have been well-documented in the literature. Flow pattern variations depending on particle size have been utilized in various microfluidic devices, which often show high efficiency [40, 41]. Besides, devices that utilize centrifugal force to induce fluid flow for the EV separation also exist [42]. However, microfluidics has limitations in terms of device size, making it challenging for industrial-scale applications due to its limited sample processing capacity. In order to operate large volume of media, the PEG precipitation method that originally employed in virus isolation processes is also utilized [43]. Because of the ease of the approach, there are also commercially available kits developed for EV isolation using PEG precipitation [44]. PEG precipitation offers a relatively short processing time but is associated with challenges such as low purity and recovery rates, as well as difficulties in removing PEG in the final step. PEG should be removed because it is an allergen-related substance [45]. To address these challenges, some studies have currently adopted the incorporation of SEC along with PEG precipitation [46]. The SEC is a chromatographic technique that uses beads with very small pores to separate particles according to their size, with larger particles eluting first. Moreover, it is a commonly used method in the separation of impurities from samples, and the isolation of EVs with this approach offers the advantage of a relatively high purity separation. Immunoaffinity is a method that uses the binding between antibodies and EVs, commonly employing antibodies specific to surface markers of EVs or specific markers from EV-secreting cells. Specific surface markers of EVs are introduced in minimal information for studies of extracellular vesicle from the International Society for Extracellular Vesicles (ISEV) [47]. Antibody-conjugated beads or columns, which exhibit reactivity with the markers, can be used for EV isolation, resulting in a high degree of EV purity. However, a limitation of this method is its lower EV yield, which poses challenges for practical application, and it typically comes with an expensive cost. In addition to the isolation methods mentioned above, there are also devices available that incorporate various characteristics of the EXODUS for EV isolation. This device could isolate EV by employing periodic negative pressure oscillations and double-coupled ultrasonic harmonic oscillations to vibrate nanoporous membranes, preventing the formation of filter cakes, similar to the TFF method, during the EV separation process. It demonstrates the advantage of producing high-purity EVs in a short period of time [48]. Finally, there is also an EV isolation method that adopts superabsorbent polymer (SAP) beads without the use of equipment or fluid flow. The SAP beads have the property of absorbing moisture and possessing micropores on their surface, enabling them to carry our self-removal with absorbing impurities [49]. In the present file of EV research, ongoing research is dedicated to exploring novel approaches for EV isolation, complementing the previously mentioned methods.

## Engineering methods of EV and applications for regenerative medicine

### Gene editing methods for EV engineering

**Fig. 3:**
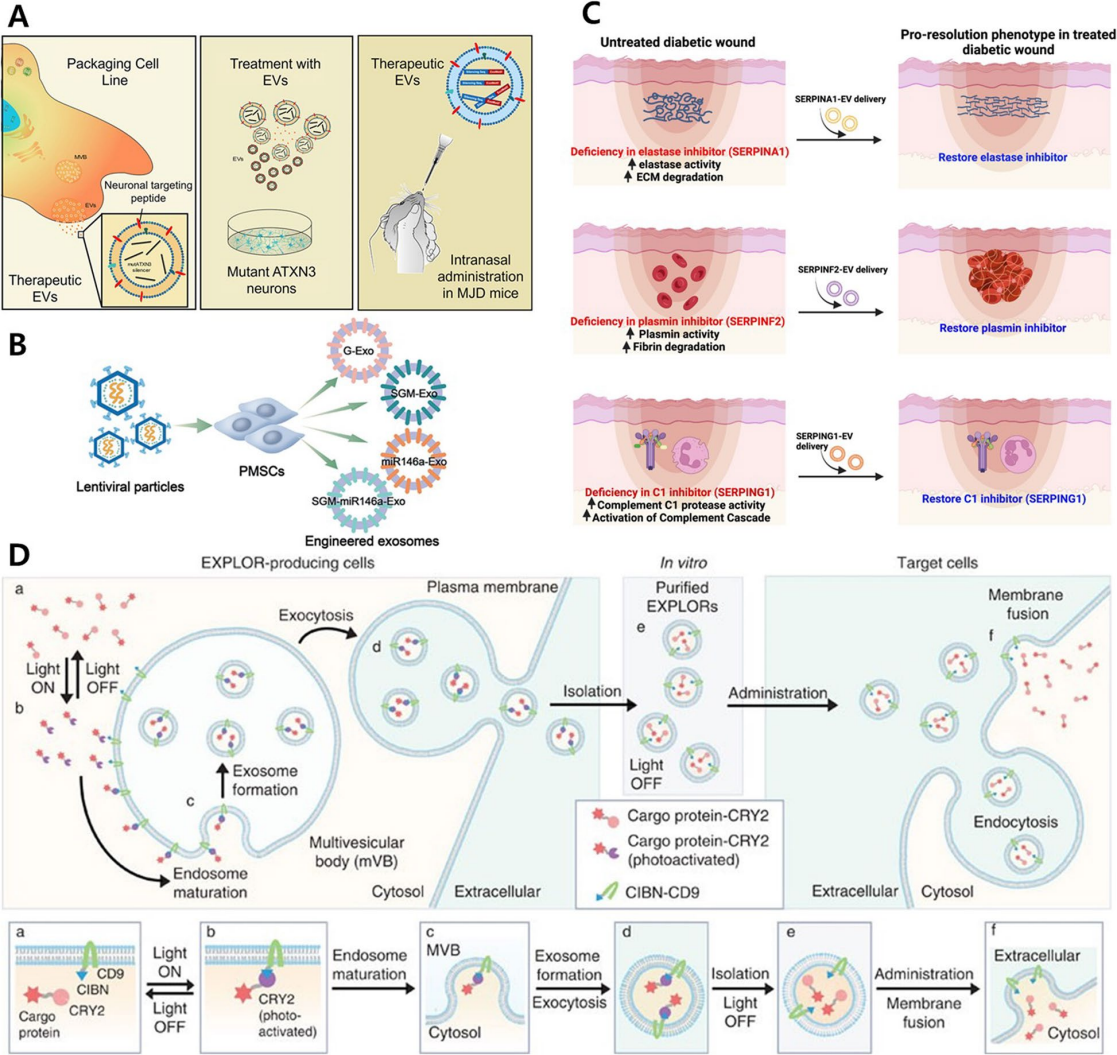
Gene editing approaches for EV engineering. **A** The ribonucleoprotein A2B1 (hnRNPA2B1) and RVG decorated EVs production using gene transfection. Reproduced with permission from [51]. Copyright 2023 Cell Press. **B** The isolation process of miR-146a encapsulated EVs. Reproduced with permission from [56]. Copyright 2023 Springer Nature. **C** The functionality of serpin overexpressed HEK293T derived EVs. Reproduced with permission from [58]. Copyright 2022 Springer Nature. **D** The schematic illustration for EXPLORs systems. Reproduced with permission from [61]. Copyright 2016 Springer Nature

**Fig. 4:**
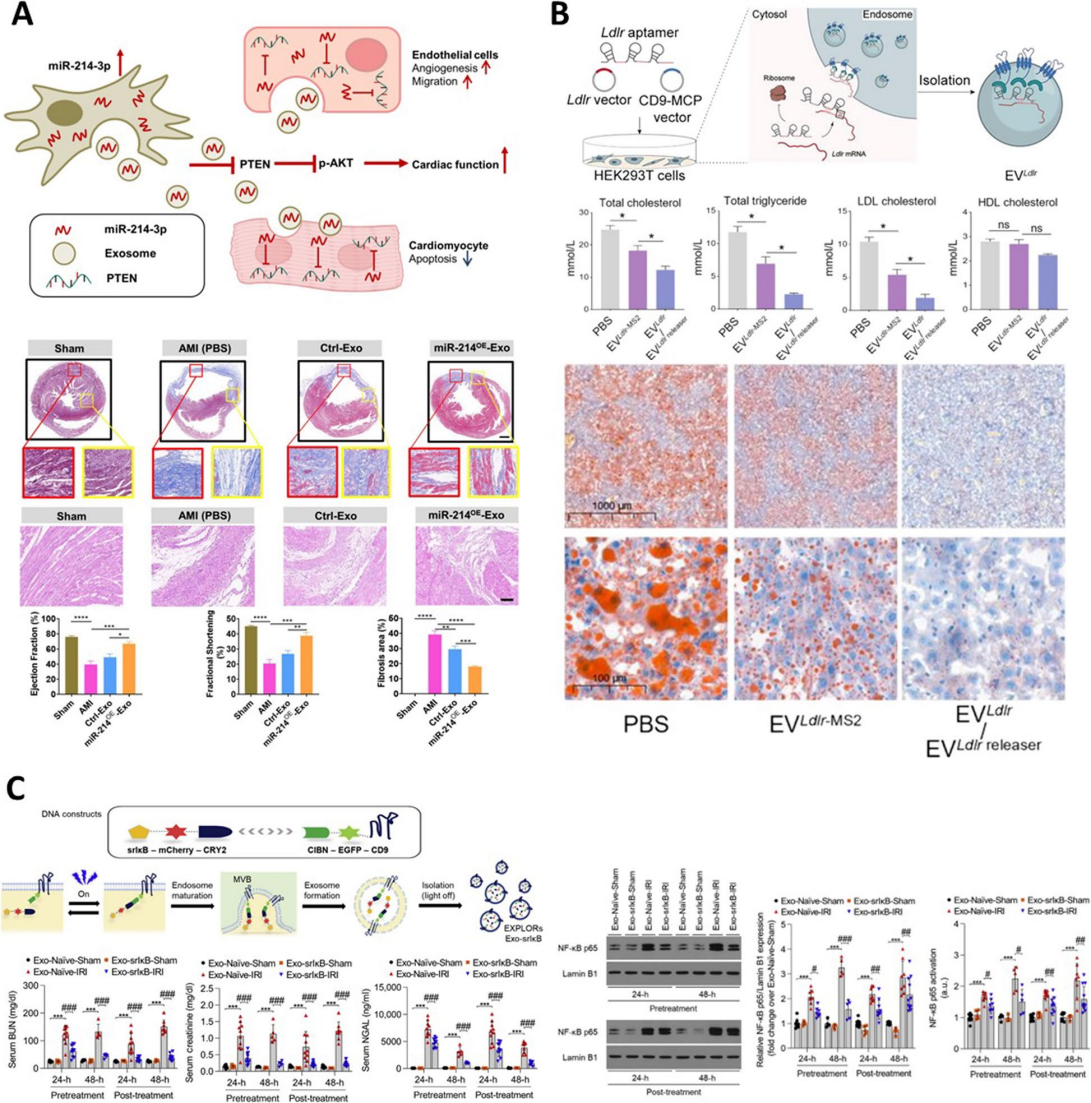
The regeneration relative applications with engineered EV through gene editing. **A** The miR-214 overexpressed EV for myocardial repair in acute myocardial infarction. Reproduced with permission from [55]. Copyright 2023 Springer Nature. **B** The Ldlr overexpressed EVs for familial hypercholesterolemia. Reproduced with permission from [60]. Copyright 2023 Ivyspring International Publisher. **C** The EXPLORs system for kidney ischemia-reperfusion injury. Reproduced with permission from [63]. Copyright 2021 Elsevier

In gene editing methods, transfection approach is commonly employed to construct systems that allow target cells to produce desired proteins/RNAs or facilitate the loading of specific factors into EVs. As indicated in Table 2, transfection agents such as Lipofectamine and the Effectene Transfection Reagent are commonly utilized for gene editing, along with lentiviruses. Target cells initially include HEK293T and HEK293, but MSCs such as PMSC and UCMSC are also employed. When treating the HEK293T cell line with lentivirus containing PDGFR-rabies virus glycoprotein (RVG), hnRNPA2B1, and mirSilencer, internalization was observed to improve through PDGFR-RVG, increased the miRNA loading due to the role of hnRNPA2B1, resulting in increased incorporation of mirSilencer (Fig. 3A) [50]. The EVs containing PDGFR-RVG, hnRNPA2B1, and mirSilencer, demonstrated an effective silencing function against ATXN3, known as the causative factor of Machado-Joseph disease (MJD) [51]. Similarly, to promote the targeting and internalization using PDGFR-RVG in this study, lentivirus was employed to express RVG and BDNF in HEK293T cells. In isolated EVs, the expression of RVG was verified and a nearly 20-fold increase in BDNF encapsulated levels was observed. In conclusion, EVs engineered to express RVG and BDNF efficiently targeted the brain when administered intranasally, promoting remyelination [52]. In the previous two studies, RVG peptide was demonstrated for effective neuronal targeting capabilities by specifically binding to acetylcholine receptors expressed in neurons [53]. Furthermore, functionalized EVs could be obtained by overexpressing miR-133-3p or miR-214-3p in UCMSC; the miR-133-3p and miR-214-3p exhibited higher internalization levels compared to control EVs, effectively modulating the AKT pathway and positively impacting cardiac function (Fig. 4A) [54, 55]. When miR-146a in human placenta was overexpressed in MSCs using lentivirus, the isolated EVs were functionally improved and demonstrated anti-inflammatory effects by suppressing IRAK1, a known upstream regulator of the inflammatory factor NF-κB, through miR-146a (Fig. 3B) [56]. When SERPINA5 or serpin proteins are overexpressed using lentivirus in HEK293 cells, the resulting EVs display efficacy by influencing the intended pathways as designed by the researchers (Fig. 3C) [57, 58]. Similarly, there are studies that the Tat protein and low-density lipoprotein receptor (Ldlr) protein were transfected into HEK293T cells using lipofectamine instead of lentivirus to functionalize EVs. The Tat protein exhibits neurotoxicity in HIV by reducing the expression levels of the p38 (synaptophysin) and the postsynaptic density 95 protein (PSD95), thus inducing synaptic damage. Researchers hypothesized that Tat protein, due to its penetrating property, could damage the cell membrane and induce neurotoxicity. In order to confirm this hypothesis, Tat-EVs without the penetrating property were produced. Upon examination, it was observed that there was no reduction in the expression levels of p38 and PSD95, which provides evidence to support the hypothesis [59].

**Table 2:** Gene editing for EV engineering

No.	Engineered materials	Method	Targeting cell	Ref.
1	PDGFR-RVG, hnRNPA2B1expressed/mirSilencer	lentivirus	HEK293T	[51]
2	RVG/BDNF	lentivirus	HEK293T	[52]
3	SERPINA5 protein	lentivirus	HEK293	[57]
4	Serpin protein	lentivirus	HEK293	[58]
5	miR-146a	lentivirus	PMSC	[56]
6	miR-214-3p	lentivirus	UCMSC	[55]
7	miR-133-3p	lentivirus	UCMSC	[54]
8	Tat protein	Lipofectamine	HEK293T	[59]
9	Ldlr mRNA	Lipofectamine	HEK293T	[60]
10	Protein drug (srIkB)	Effectene Transfection Reagent	HEK293T	[61]
11	Protein drug (srIkB)	Effectene Transfection Reagent	HEK293T	[62]
12	Protein drug (srIkB)	Effectene Transfection Reagent	HEK293T	[63]

Ldlr is a lipoprotein receptor located on the surface of hepatocytes, and when it fails to function properly, it can lead to metabolic disorders such as Familial Hypercholesterolemia (FH). In this study, administering Ldlr-EVs as a treatment to mice lacking Ldlr expression resulted in an improvement in lipid metabolism (Fig. 4B) [60]. The previously mentioned method of transfecting cells to produce functionalized EVs has also been applied to create a platform technique to load protein drugs. The platform called as exosomes for protein loading through optically reversible protein–protein interactions (EXPLORs) was fabricated with HEK293T cells that were transfected to express the CIBN-EGFP-CD9 protein (Fig. 3D). The engineered cells were supplemented with free Cry2-tagged cargo proteins within the culture media, and then the activation of Cry2 and CIBN binding was achieved by exposing them to 488 nm light. The Cry2-CIBN system serves as a platform technology for the efficient loading of a variety of protein drugs into EVs, offering the advantage of versatility [61]. The cargo protein selected for loading into EVs was the potent NF-κB inhibition using this technology, known as the super-repressor IκB (srIκB), resulting in the production of EVs loaded with srIκB. The efficacy of srIκB-loaded EVs was verified through in vitro and in vivo experiments, which confirmed their effective inhibition of NF-κB (Fig. 4C) [62]. This technology has been in Phase 1 clinical trials in Australia, and no serious adverse events (AEs) or adverse drug reactions (ADRs) were reported, confirming its safety. Based on these results, it can be anticipated that loading various types of protein drugs into EVs can be safely applied to treat a wide range of diseases. Researchers believe that the use of gene editing for EV engineering technology will become a valuable tool in the near future.

### Endogenous methods for EV engineering

#### Inflammatory factor preconditioning for EV functionalization

**Fig. 5:**
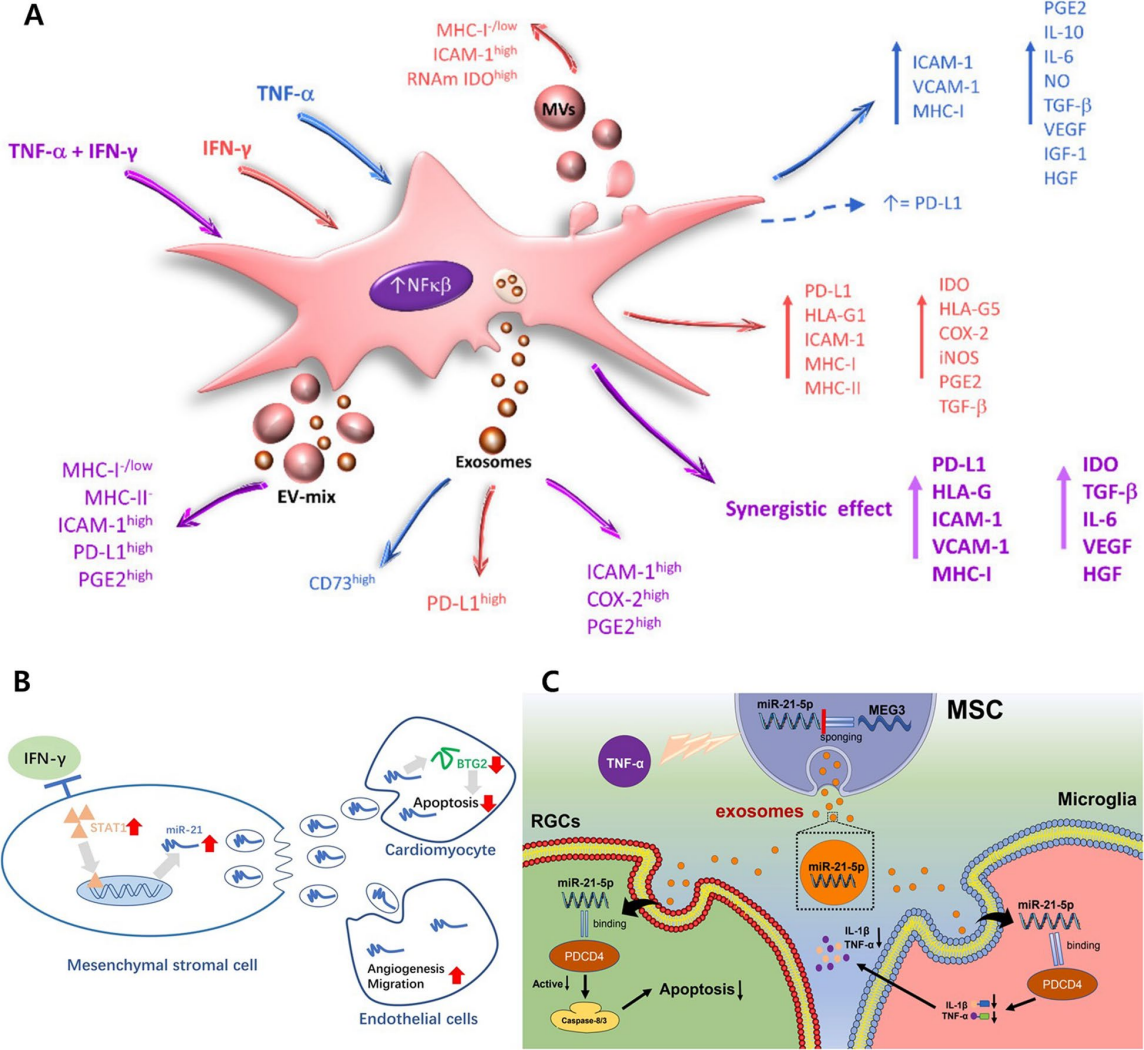
Endogenous modification methods for EV engineering using inflammation related factors. **A** The activation and functional changes of MSC preconditioned with inflammation related factors. Reproduced with permission from [14]. Copyright 2021 MDPI. **B** The mechanism of functional enhancements of MSC-derived EV preconditioned with INF-γ. Reproduced with permission from [65]. Copyright 2022 Springer Nature. **C** The TNF-α preconditioning effects on the process of MSC-derived EV production. Reproduced with permission from [66]. Copyright 2022 Elsevier

Endogenous EV engineering can be categorized into two main approaches, the inflammation factor and non-inflammation related factors preconditioning. First, inflammation factor preconditioning is a well-known method employed in utilizing MSC (Fig. 5A). When treated with inflammatory factors such as TNF-α or IFN-γ, it is known that the secreted EVs exhibit enhanced immunomodulatory, regenerative, angiogenic, anti-apoptotic, and anti-scarring effects, along with the cells themselves [64]. The increases in these effects are attributed to MSC activation induced by inflammatory factors, and this mechanism has been leveraged in numerous studies [14]. The EVs derived from INF-y treated UCMSCs, as commonly known, improved wound healing, angiogenesis, anti-inflammatory and anti-apoptosis effects, and miR-21-5p levels were found to be increased compared to the control EVs (Fig. 5B). The increase in miR-21-5p levels has been elucidated to be the result of activation of the STAT pathway following INF-y treatment of MSCs. In cardiomyocytes, this led to the inhibition of BTG2 expression, thus suppressing apoptosis. In endothelial cells, it enhanced angiogenesis and migration effects, ultimately improving heart function and reducing the size of the infarct, [65] on the other hand, when treated exclusively with TNF-α, GMSC-derived EVs exhibited increased antiapoptotic and anti-inflammatory effects, similar to the discussed earlier (Fig. 5C). Furthermore, they also showed an increase in the loading of miR-21-5p. The action of miR-21-5p was demonstrated to have anti-apoptotic effects in retinal ganglion cells (RGCs) and anti-inflammatory effects in microglia [66]. Similar to individual preconditioning of TNF-α or IFN-γ, both factors in combination (TNF-α and IFN-γ; TI) can notably induce MSC activation. Treatment of BMSCs with TNF-α (15 ng/ml) and IFN-γ (10 ng/ml) displayed immune modulation effects on immune effector cells (IECs), including T, B, and NK cells. Although some of these effects were observed with native EV, treatment with inflammation factors resulted in more pronounced and enhanced effects [67]. When these EVs were applied to inflamed primary rat splenocytes, they led to a mechanism of COX2/PGE2 and exhibited an anti-inflammatory effect [68]. This anti-inflammatory capacity was also augmented in EVs derived from UCMSCs. In particular, this effect was most prominent in EV isolated after treating the cells with TI (20 ng/ml; 20 ng/ml) in a 3D culture environment compared to a 2D culture system. Through bioinformatics analysis, it was determined that changes in the EV characteristics in response to environmental alterations included anti-inflammatory effects and angiogenesis, wound healing, anti-apoptosis, anti-fibrosis, etc. [69].

**Fig. 6:**
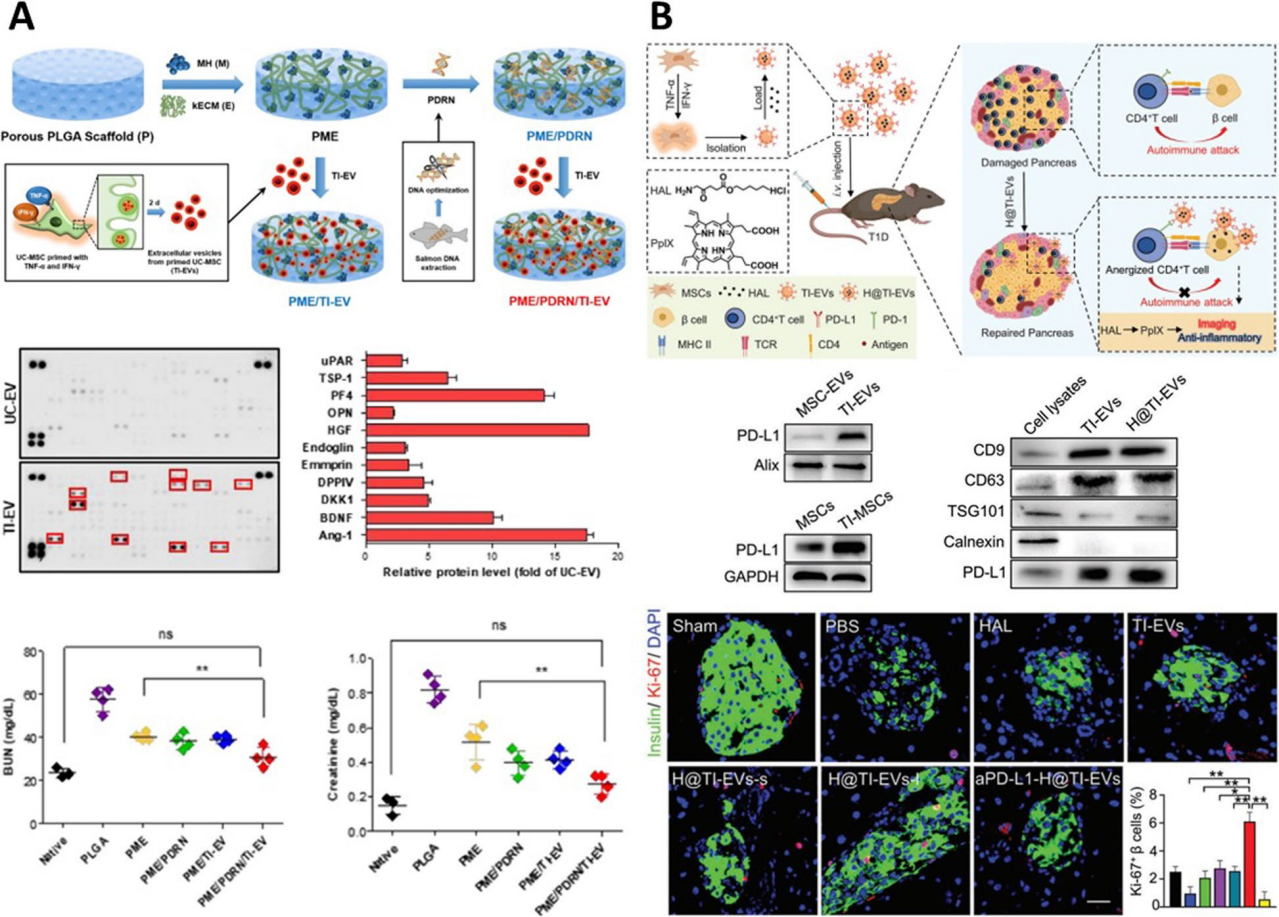
Various applications on regenerative medicine using endogenous engineered EVs. **A** The functional enhancements with TNF-α and INF-γ (TI) priming on MSC and the therapeutic effects on chronic kidney disease (CKD). Reproduced with permission from [21]. Copyright 2021 American Chemical Society. **B** High expression of PD-L1 and applications for immunotherapy in type 1 diabetes with TNF-α and INF-γ preconditioned MSC derived EV (TI-EV). Reproduced with permission from [71]. Copyright 2023 John Wiley & Sons

Various regenerative functions of TI-primed UCMSC EV can be applied to chronic kidney disease (CKD) (Fig. 6A). Additionally, it was shown to have an M1/M2 polarization effect. As a result of glomerular numbers increased, glomerular filtration rate (GFR) values were restored to levels similar to the control group, and BUN and creatinine levels decreased, contributing to renal regeneration [21]. The M1/M2 polarization effect in UCMSC is enhanced after TI priming, and this effect appears to be due to increased expression of PD-L1 in TI-primed UCMSC EV [70]. Additionally, using these characteristics, TI-primed UCMSC EV with increased PD-L1 expression can suppress CD4+ T cells, preventing cell destruction of β cells and demonstrating therapeutic effects in Type 1 diabetes (T1D), an autoimmune disease (Fig. 6B) [71]. The M1/M2 polarization effect can also be facilitated by EVs derived from GMSCs treated with TNF-α (50 ng/ml) and IFN-α (50 ng/ml), due to an increase in the expression levels of CD73 and CD5L [72]. BMSCs primed with another inflammatory factor, IL-1β (10 ng/ml), derived EVs exhibited potent anti-inflammatory effects, as well as characteristics such as wound healing and angiogenesis, demonstrating regenerative effects in endometritis [73]. Furthermore, IL-1β-primed BMSC EVs modulated the SIRT1/ERK pathway, showing anti-inflammatory and anti-apoptotic effects, thus improving stress of the septic endoplasmic reticulum [74]. Treatment of BMSC with another inflammation factor, lipopolysaccharides (LPS; 100 ng/ml), also improved the functionality of EVs, resulting in accelerated functional recovery, axon regeneration, remyelination, and M1/M2 macrophage polarization in a rat sciatic nerve injury model. These regenerative effects are attributed to the abundant presence of TNF-stimulated gene-6 (TSG-6) within LPS-preconditioned BMSC EVs. TSG-6 has been shown to accelerate the process by inhibiting NF-κB and NOD-like receptor protein 3 (NLRP3) [75]. The LPS-preconditioned BMSC EVs also demonstrated regenerative effects in septic liver injury, which is attributed to an increased expression of autophagy-related protein 2 homolog B (ATG2B) in LPS-preconditioned BMSC EVs compared to native EVs. Activation of ATG2B upregulated mitophagy in intrahepatic macrophages inhibits the release of mtDNA into the cytosol and consequently suppresses macrophage stimulator of interferon genes (STING) signaling. As demonstrated in previous research, STING signaling, a well-established therapeutic target in numerous inflammatory conditions, could also influence septic liver injury [76]. The EVs derived from numerous preconditioned MSCs with inflammatory factors exhibited significant changes in their properties, presenting enhanced regenerative effects with multiple pathways such as anti-inflammation, anti-fibrosis, and M1/M2 macrophage polarization, in contrast to non-preconditioned control EVs (Table 3).

**Table 3:** Cell preconditioning with inflammatory factors for EV engineering

No.	Target cell	Treatment material	Concentration	Ref.
1	UCMSC	IFN-γ	50 ng/ml	[65]
2	GMSC	TNF-α	10 ng/ml	[66]
3	BMSC	TNF-α, IFN-γ	15 ng/ml; 10 ng/ml	[67]
4	BMSC	TNF-α, IFN-γ	20 ng/ml; 20 ng/ml	[68]
5	UCMSC	TNF-α, IFN-γ	20 ng/ml; 20 ng/ml	[69]
6	UCMSC	TNF-α, IFN-γ	20 ng/ml; 20 ng/ml	[21]
7	UCMSC	TNF-α, IFN-γ	20 ng/ml; 20 ng/ml	[70]
8	GMSC	TNF-α, IFN-α	50 ng/ml; 50 ng/ml	[72]
9	BMSC	IL-1β	10 ng/ml	[73]
10	BMSC	IL-1β	10 ng/ml	[74]
11	BMSC	LPS	100 ng/ml	[75]
12	BMSC	LPS	100 ng/ml	[76]

#### Non-inflammation related factors preconditioning for EV functionalization

**Fig. 7:**
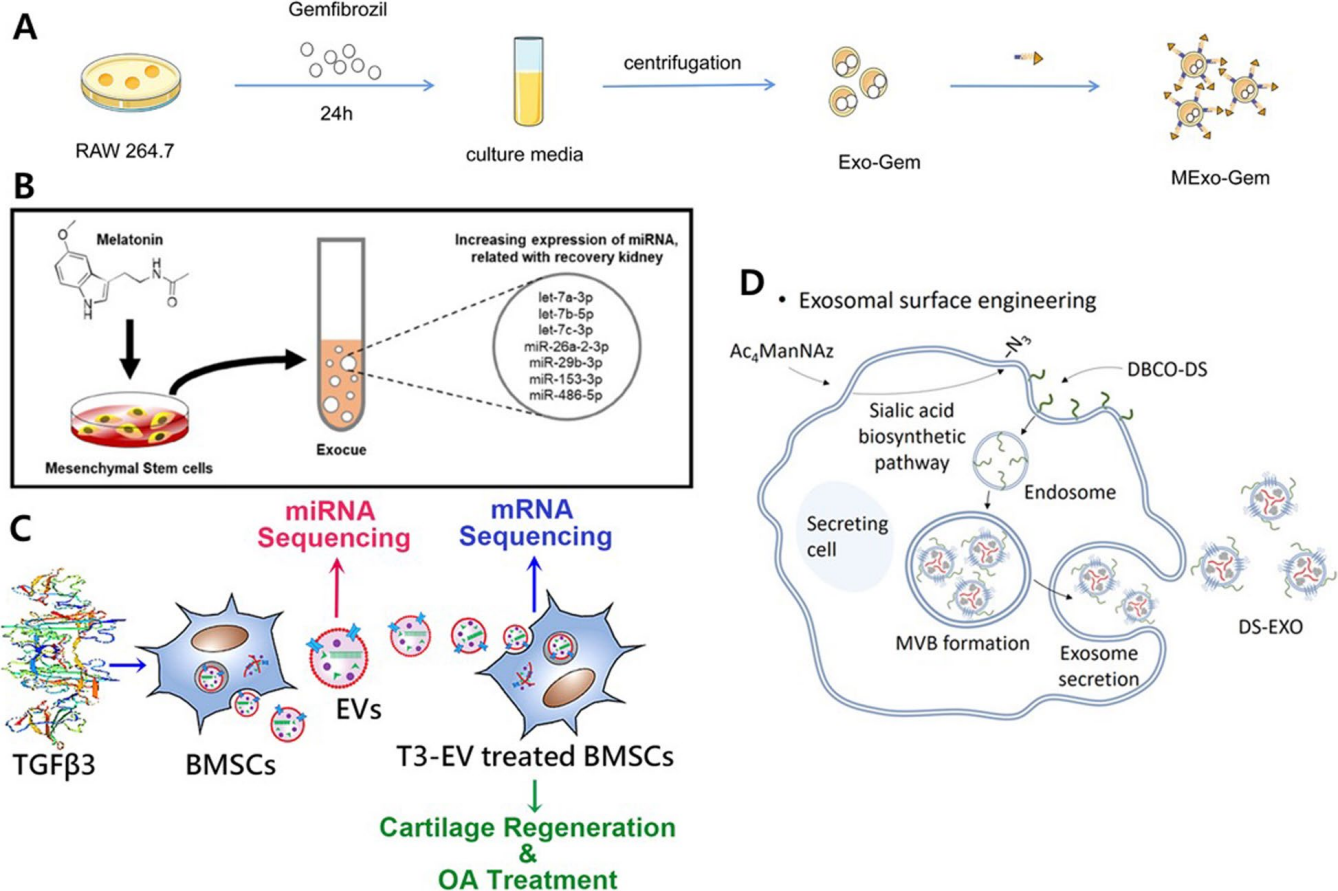
Endogenous modification methods for EV engineering using non-inflammation related factors. **A** The production process of EVs derived from gemfibrozil preconditioned RAW264.7 and their therapeutic pathway for AD. Reproduced with permission from [77]. Copyright 2022 Elsevier. **B** The functional enhancement and the production process of melatonin preconditioned ADMSC derived EV. Reproduced with permission from [87]. Copyright 2021 SAGE Publications. **C** The production process of EV derived from cartilage differentiation process with TGF-β3. Reproduced with permission from [15]. Copyright 2022 Springer Nature. **D** The surface engineering method for EVs using click chemistry with N-azidoacetylmannosamine-tetraacetylated (Ac4ManNAz) treatments. Reproduced with permission from [92]. Copyright 2021 AAAS

Engineering approaches have also been used to enhance the characteristics of EVs using drugs, peptides, and growth factors as preconditioning agents, rather than inflammatory factors. The Gemfibrozil (Gem)-loaded EVs could be isolated by treating Raw 264.7 cells with Gem, and these Gem-EVs were applied to Alzheimer’s disease (AD), where impaired intracellular clearance and degradation of Aβ are considered as contributing factors (Fig. 7A). The Gem-EVs were administered via intraperitoneal (i.p.) injection and later exhibited the ability to upregulate the intracellular degradation and clearance of Aβ. This effect was achieved through the induction of PPARα-mediated nuclear translocation of TFEB, which consequently activated lysosomes for enhanced Aβ intracellular degradation and clearance in microglia as key immune cells. As a result, it was possible to improve the learning and memory abilities in the AD mouse model [77]. Similarly, there is research using silibinin (Slb), which has the effect of reducing Aβ aggregation and improving patients’ behavior and cognitive abilities. The Slb possesses the potential to improve AD, which has the drawbacks related to low brain targeting capabilities and limited bioavailability. In order to overcome these limitations, this study sought to address these drawbacks by loading Sib into EVs, creating Sib-EVs. As a result, Slb-EV exhibited the capacity to selectively bind to Aβ, preventing its aggregation, inhibiting astrocyte activation, and reducing the secretion of proinflammatory cytokines. The Slb-EV also regulated the NF-κB pathway, reversing neuronal damage and improving cognitive function in an Aβ-induced AD model [78]. The Edaravone (Edv), a drug clinically used for stroke, shows a short half-life and low bioavailability like Slb, prompting its encapsulation within EVs (Edv-EV) to address these limitations. The Edv-EV derived from RAW 264.7 cells exhibited significant alterations in pharmacokinetic parameters, leading to increased bioavailability. Additionally, they efficiently delivered Edv to the ischemic brain in the permanent middle cerebral artery occlusion (pMCAO) model, demonstrating neuroprotective effects [79]. The Cystatin C (CysC), associated with neuroprotection and recovery in the nervous system, can be directly secreted in its soluble form by cells and can also be encapsulated within EVs for secretion. When comparing EVs derived from CysC-deficient cells and CysC-EVs isolated through CysC treatment, CysC-EVs showed higher levels of secretion from cells and demonstrated a positive effect on cell survival than control EVs [80]. The Hemin (Hem), known to induce HO-1 expression in various cell types and then regulate the anti-oxidation and the anti-inflammation, was reported to increase HO-1 when administered to dendritic cells (DCs). Based on these effects, when Hem treated DC-derived treated EVs (Hem-EV) were administered via inhalation to a house dust mite (HDM) induced asthmatic mouse model, it reduced eosinophil infiltration and mucus secretion in the airways, decreased the levels of IL-4, IL-5, and IL-13 in the lungs, and reduced the Th2 cells number in the mediastinal lymph nodes (MLNs), while increasing the number of regulatory T (Treg) cells in the MLNs. These immune-regulatory effects are believed to be attributed to the regulation of HO-1 expression in Hem-EVs [81]. The resveratrol (Res), a well-known antioxidant compound and non-flavonoid polyphenol, was successfully loaded into EV derived from primary microglia. In this form, Res demonstrated higher stability and a delayed degradation rate compared to those in its soluble form. EV derived from Res preconditioned primary microglia induced the activation of the PI3K signaling pathway, promoting neuronal survival, inhibiting apoptosis, improving autophagy, and enhancing motor function in a mouse model with spinal cord injury (SCI) [17]. When the erythropoietin (EPO) hormone, a protein that stimulates the formation and differentiation of red blood cells, was applied to BMSCs, it was observed that the number of particles increased in a dose-dependently within the range of 1 to 100 IU/ml. Additionally, EPO-treated BMSC-derived microvesicles (EPO-MVs) displayed an inhibitory effect on TGF-β1-induced fibrosis [82]. When investigating and analyzing the miRNA profiles of EPO-MVs and control-MVs, it was found that EPO-MVs exhibited a relatively upregulated expression of microRNAs known to inhibit fibrosis, such as miR-302 and miR-200. As a result, in an in vivo study, a reduction in fibrosis-associated markers expression levels, including α-SMA and E-cadherin, was observed. This suggests an anti-fibrosis effect in a mouse model of CKD that shows improvements in the levels of BUN and serum creatinine [83, 84]. When Tanshinone IIA (TSA), an effective drug for myocardial ischemia-reperfusion injury (MI/RI), was treated with UCMSC and its EVs were isolated and delivered into the myocardium in vivo, it demonstrated an effect of reducing the size of the infarct while improving heart function, compared to non-treated group. These effects were validated by the CCR2 suppression effect of EV on CCR2^+^ macrophages, known to promote inflammation similar to M1 macrophages. In the MI/RI mouse model, when treated with TSA-treated UCMSC-derived EVs (TSA-EV) and control-EV, the TSA-EV-treated group exhibited a reduction in the number of CCR2^+^ macrophages, and a significant decrease in CCR2 mRNA expression was observed. Furthermore, in the examination of the miRNA content of TSA-EV and control-EV, it was observed that TSA-EV contained a higher level of miR-223-5p, which was found to reduce the expression of CCR2, leading to the suppression of the infiltration of circulating monocytes that promotes inflammation. It was confirmed through a decrease in the markers CD68 for macrophages and CD11b^+^ for monocytes. Moreover, the decrease in CCR2 expression was associated with an increase in the expression of α-SMA, VEGF, and CD31 expression, indicating enhanced angiogenesis [85]. Similar to TSA-EV demonstrating regenerative effects, melatonin (Mel), known for its role in the regulation of neuroendocrine and immune system interactions, has exhibited various regenerative effects when utilized in Mel-treated MSC-derived EVs (Mel-EV, Fig. 7B) [86]. The level of let-7b-5p, miR-23a-3p, and miR-100-5p levels were higher in the Mel-EV derived from the UCMSC under the XFM condition than under the starvation condition. Bioinformatics analysis revealed that these changes were associated with the promotion of anti-apoptosis and cell migration. Based on these analytical results, researchers substantiated the potential for regenerative effects within the kidneys by conducting experiments on HK2 cells, a type of proximal tubule epithelial cell, to validate characteristics such as angiogenesis, wound healing, anti-inflammation, ROS scavenging, and anti-apoptosis [16]. In a comparison of Mel-EV derived from ADMSC with control EV, the upregulation of several miRNAs was observed. Upon administration to a mouse model of CKD, the Mel-EV group showed relatively lower expression levels of inflammation and fibrosis-related factors such as TGF-β, NF-κB, IGF-1, and CTGF compared to the control group. In renal tissues from the Mel-EV group, factors associated with water absorption, AQP2 and AQP5, were detected at relatively higher levels. Furthermore, BUN and creatinine levels showed improvements comparable to those in healthy mice [87]. When comparing control-EV and Mel-EV derived from ADMSCs with miRNA sequencing, an increase in the expression level of miR-10a-3p was observed in Mel-EV. The miR-10a-3p was found to be highly expressed not only in Mel-EV but also in Mel-treated ADMSCs and Mel-EV-treated cavernosum smooth muscle cells (CSMCs). TargetScan analysis (TargetScan v 8.0, https://www.targetscan.org/) identified protein kinase inhibitor α (PKIA) as a target gene of miR-10a-3p. In addition, in an analysis of CSMC expression levels, PKIA was observed to be reduced in the Mel-EV treatment group. The relation between miR-10a-3p and PKIA and their connection to the RhoA/ROCK pathway, which is closely related to the functions of CSMC, was investigated. This analysis was based on previous reports on the transduction of the PKIA/RhoA/ROCK signal in endothelial cells [88, 89]. As a result, it was evident that Mel-EV could effectively suppress the phenotypic regulation of CSMC through the miR-10a-3p/PKIA/RhoA/ROCK axis, leading to significant improvement in cavernous nerve injury erectile dysfunction (CNI ED) [90]. Analysis of selenium, a known factor that influences MSC proliferation, multipotency, and anti-inflammatory effects, treated ADMSC-derived EV (Sel-EV), produced results like those of EV characteristics regulated by the previously mentioned substances. These results indicated effects such as wound healing, angiogenesis, and anti-inflammation [91].

**Fig. 8:**
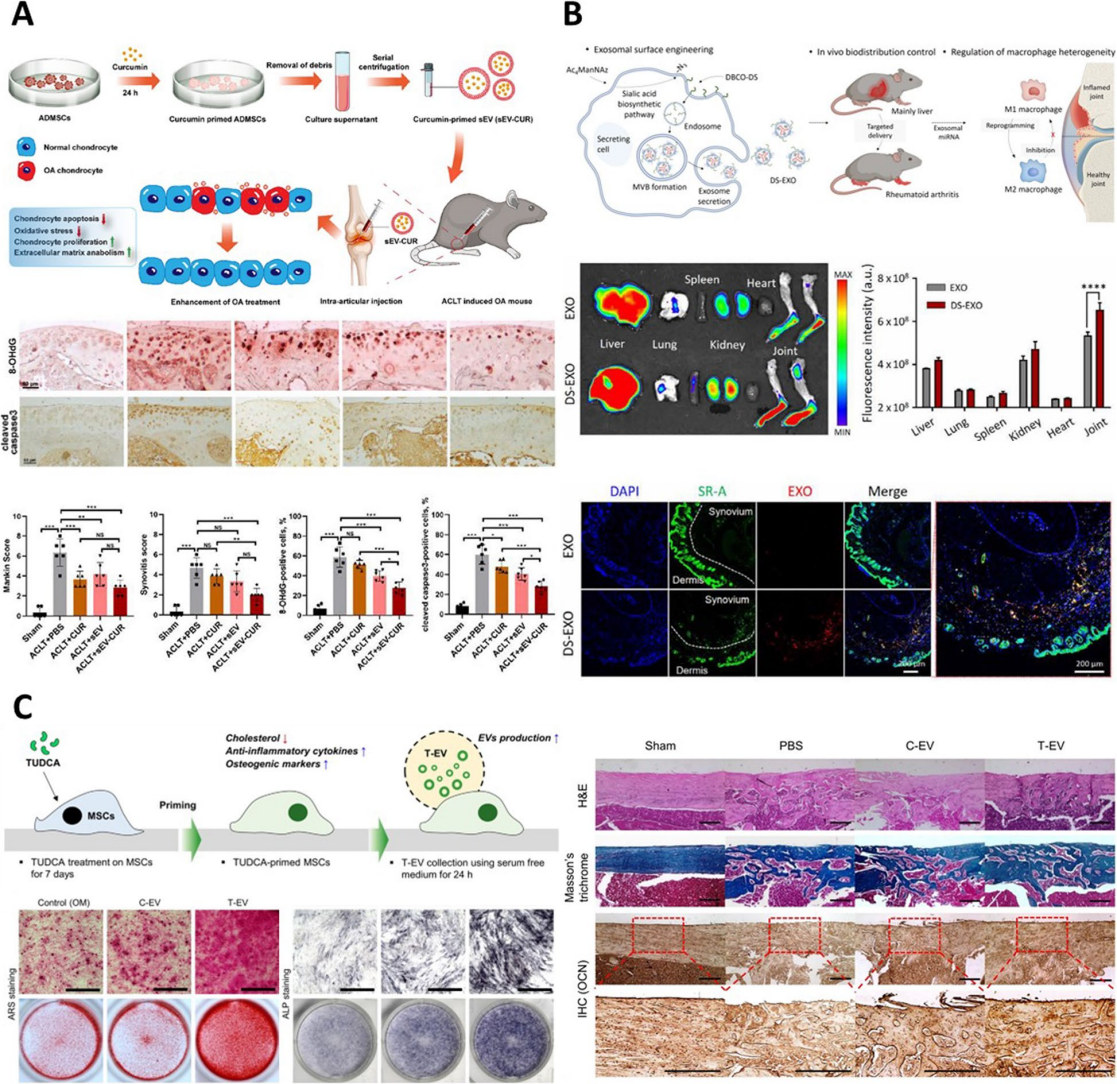
Various applications on regenerative medicines using endogenous engineered EV with non-inflammatory factors. **A** The therapeutic effect on osteoarthritis (OA) using curcumin preconditioned ADMSC-derived EV. Reproduced with permission from [18]. Copyright 2022 Springer Nature. **B** The surface engineering of EVs with Ac4ManNAz preconditioning and their immunotherapeutic effect on rheumatoid arthritis (RA). Reproduced with permission from [92]. Copyright 2021 AAAS. **C** Anti-inflammatory and osteogenic activities of the tauroursodeoxycholic acid (TUDCA) preconditioned BMSC-derive EVs. Reproduced with permission from [95]. Copyright 2023 Elsevier B.V

Furthermore, when Cur, known for its excellent antioxidant effects, was used to isolate Cur treated ADMSC derived EV (Cur-EV), an increase in cell proliferation was observed in a model of chondrocyte OA model induced by tert-Butyl hydroperoxide (TBHP, Fig. 8A). Additionally, increases in factors associated with cartilage repair, including aggrecan (ACAN) and COL II, and the inhibition of factors related to cartilage damage, such as ADAMTS5, MMP13, IL-1β, and TNF-α were observed. Cur-EV also shows antioxidant and anti-apoptotic effects. These effects were observed in an anterior cruciate ligament transection (ACLT)-induced mouse model, confirming the prevention of cartilage damage and the regenerative potential [18]. In addition to engineering EVs by treating cells with beneficial compounds, another approach involves utilizing EVs secreted by cells during the MSC differentiation. Researchers obtained EVs secreted during the period of chondrogenic differentiation by treating BMSCs with TGFβ3 and then examined their therapeutic effects on OA models (Fig. 7C). When treated with TGFβ3-treated BMSC-derived EVs (T3-EVs), BMSCs exhibited an increase in the expression levels of chondrogenic markers, such as SOX9, ACAN, glycosaminoglycan (GAG), collagen I (COL1), and collagen II (COL2), similar to those treated with TGF-β3. Subsequently, miRNA sequencing was conducted on BMSCs treated with T3-EVs. This result enabled the identification of high expression of miRNA-455-5p in T3-EVs and the regulation of FOXO signaling in T3-EV-treated BMSCs. Subsequently, miRNA-455-5p was identified as targeting the SOX11 gene using bioinformatic analysis. Therefore, these findings suggested that the presence of SOX11 suppresses the expression of SOX9 and FOXO1 during chondrogenic differentiation. By inhibiting SOX11 with miRNA-455-5p, normal chondrogenic differentiation could be activated, offering a potential pathway for the treatment of OA [15]. Finally, the endogenous engineering approach also involves treating cells with substances to induce the expression of functional groups on the surface of secreted EVs. It can be achieved by treating cells with N-azidoacetylmannosamine-tetraacetylated (Ac4ManNAz), and EVs secreted by Ac4ManNAz-treated cells exhibit the expression of azide groups on their surface (Fig. 7D) [93]. The azide groups present on these EVs could be used for attaching dibenzocyclooctyne-conjugated dextran sulfate (DBCO-DS) through click chemistry, resulting in the generation of DS-EVs. DS can target macrophage scavenger receptor class A (SR-A), which is commonly observed in the inflammatory joint environment of rheumatoid arthritis (RA, Fig. 8B) [94]. The targeting function of DS-EV was demonstrated through fluorescence imaging, which showed higher intensity in the joint area compared to control EV. Additionally, the DS-EV group exhibited a relatively reduced expression of SR-A. In fact, in vivo results confirmed that DS-EV played a role in the induction of complex regenerative effects, including M1/M2 polarization, and improvements in RA-related factors [92]. In bone regeneration, tauroursodeoxycholic acid (TUDCA) could be utilized to regulate stem cell differentiation into osteogenesis and prevent adipogenic differentiation. To increase production yield and function of MSC-EVs, TUDCA has been treated to MSC and bone tissue regeneration-related bioactivities of TUDCA pretreated MSC-derived EVs (T-EVs) were investigated with incorporation into the collagen scaffold (Fig. 8C). T-EV facilitated osteogenic differentiation of MSC. Furthermore, bone formation and the release of anti-inflammatory cytokine were achieved with in vivo transplantation of T-EV compared to control EVs [95]. Similarly, due to angiogenic properties of dimethyloxaloylglycine (DMOG), the angiogenesis of tissue-engineered bone and bone healing abilities were improved with low dose of DMOG-pretreated MSC derived EVs (DMOG-MSC-Exos) [96]. Bone regeneration and angiogenesis were enhanced in a critical-sized calvarial defect rat model with DMOG-MSC-Exos via AKT/mTOR pathway, which is known to be the major signaling pathway for the proangiogenic activities. In summary, endogenous engineering approaches aim to treat cells with beneficial substances or the desired encapsulation of substances into cell-derived EVs. Alternatively, they may induce the expression of specific functional groups on the surface of EVs. This engineering approach can be utilized to enhance drug stability and improve their delivery efficiency to target sites and is considered a versatile technology applicable to various diseases (Table 4).

**Table 4:** Cell preconditioning with non-inflammation related factors for EV engineering

No.	Target cell	Treatment material	Concentration	Ref.
1	RAW 264.7	Gemfibrozil	100 μg/ml	[77]
2	RAW 264.7	Silibinin	50 μg/ml	[78]
3	RAW 264.7	Edaravone	87.5 μg/ml	[79]
4	SMC	Cystatin C	1.95 μg/ml	[80]
5	Dendritic cell	Hemin	4.89 μg/ml	[81]
6	Primary microglia and neurons	Resveratrol	9.13 μg/ml	[17]
7	BMSC	TGFβ3	50 ng/ml	[15]
8	BMSC	Erythropoietin (EPO)	0.84 μg/ml	[82]
9	BMSC	tauroursodeoxycholic acid (TUDCA)	1.3 mg/ml	[95]
10	BMSC	Dimethyloxaloylglycine (DMOG)	0.17 mg/ml	[96]
11	UCMSC	Tanshinone IIA	2.94 μg/ml	[85]
12	UCMSC	Melatonin	4.65 μg/ml	[16]
13	ADMSC	Melatonin	4.65 μg/ml	[87]
14	ADMSC	Melatonin	2.32 ng/ml	[90]
15	ADMSC	Selenium	5 ng/ml	[91]
16	ADMSC	Curcumin	3.68 μg/ml	[18]
17	ADMSC	Ac4ManNAz	8.61 μg/ml	[92]

### Exogenous methods for EV engineering

Exogenous EV engineering involves subjecting previously isolated EVs to various external forces, enabling the penetration of desired substances through their membranes into the EV’s interior. The simplest method involves incubating the substance for encapsulation within EVs in an EV solution, facilitating its diffusion into the interior of EVs [97–100]. Coenzyme Q10 (CoQ10), known for its anti-inflammatory and antioxidant properties, is a promising substance for its potential effectiveness in AD [101]. A common challenge associated with substances that exhibit therapeutic effects in AD is their difficulty in penetrating the blood-brain barrier (BBB) and, consequently, their limited ability to directly reach the affected brain regions of the brain. In order to address this problem, the study involved loading CoQ10 into ADMSC-derived EVs, known for their non-immunogenicity and containing neprilysin (NEP) as an Aβ-degrading enzyme, resulting in CoQ10-EVs [102]. Then, their effects in an AD rat model induced by streptozotocin (STZ). The results of the Morris water maze (MWM) test in animals showed improvements among the CoQ10, EV, and CoQ10-EV treated groups compared to the AD model group. In particular, the CoQ10-EV group exhibited the most significant improvement. Furthermore, the CoQ10-EV group exhibited elevated levels of SOX2, a factor known to inhibit the loss of neurons and neural stem cells in the hippocampus, the region associated with memory. The level of BDNF, typically highly expressed in AD, also decreased in the CoQ10-EV group, unlike the AD model group. These results suggest that the administration of the form of CoQ10-EV yields a more significant improvement compared to the administration of the drug or EV alone [103]. To load a larger amount of substances more efficiently into EVs, active incubation methods utilizing surfactants such as saponin or Triton are used, which dissolve the membrane components to create nanopores. Another method involves the use of freeze and thaw cycles, alternating between freezing and room temperature conditions, to destabilize the membrane, allowing substance loading [104–106]. The 0.2% saponin and bevacizumab (BZ) in the same proportion as the EV proteins were incubated for 30 min, active incubation method, to load BZ into the EVs. Repeated cycles of freezing at − 80 °C for 30 min. and thawing process, freeze and thaw method, were also performed to facilitate the loading of BZ into EVs. Despite these methodological differences, it was noted that there were no significant differences in the loading efficiency when compared to the incubation method. In general, it was observed that almost 30% of the drug was loaded into the EVs. The produced BZ loaded EVs (BZ-EVs) retained the characteristic anti-angiogenic properties of BZ and demonstrated the ability to suppress VEGF levels in diabetic retinopathy [107]. Additionally, the extrusion method is also utilized to introduce drugs into EVs by repetitively passing them through a polycarbonate membrane with uniform-sized pores. In order to utilize this method, neutrophil-derived EVs (N-EVs) were first isolated and a certain amount of doxorubicin (DOX) was dissolved in the N-EV solution. Subsequently, the EVs were uniformized through a mini extruder, successively passing through polycarbonate membrane filters with pore sizes of 1 μm, 400 nm, and 200 nm, facilitating drug loading [108]. Similarly, EVs derived from UCMSC were co-extruded with paclitaxel (PTX) for drug loading. Compared to the previously mentioned freeze and thaw method, the extrusion method demonstrated an approximately 14% drug loading efficiency, whereas the freeze and thaw method exhibited around 7%. Subsequently, the migration of neural stem cells into the scaffold was observed when PTX-loaded EVs produced in this manner were integrated into a collagen scaffold and applied to an SCI rat model. This was evident through the increase in markers such as Tuj-1 and Map2, which indicates functional neural regeneration [109].

**Fig. 9:**
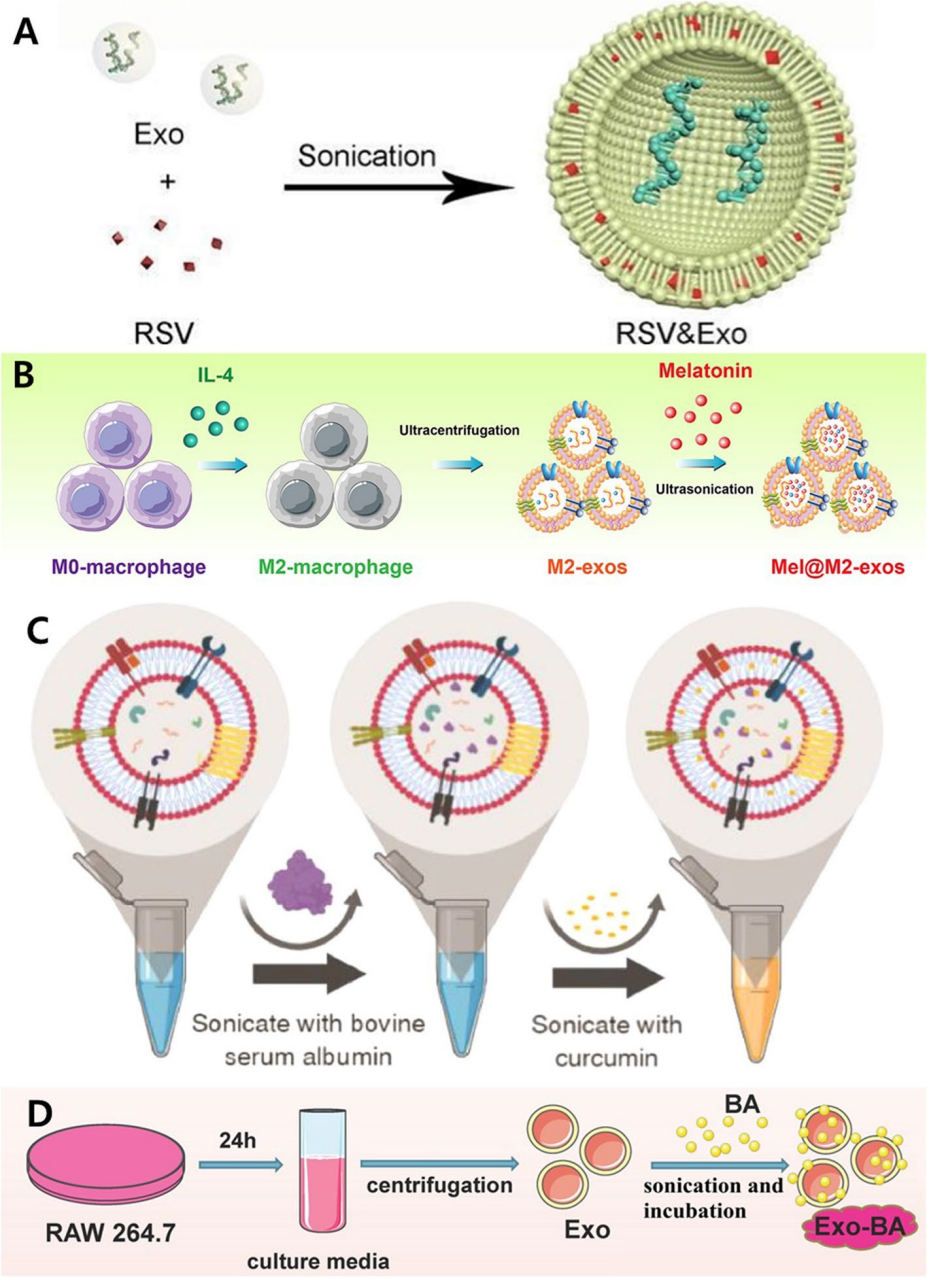
Exogenous modifications for EV engineering. **A** The resveratrol encapsulated EVs fabricated with sonication method. Reproduced with permission from [113]. Copyright 2023 Elsevier. **B** The melatonin (Mel) integrated M2 macrophage-derived EV produced with sonication. Reproduced with permission from [114]. Copyright 2023 John Wiley & Sons. **C** The curcumin loaded EVs fabricated using sonication method. Reproduced with permission from [115]. Copyright 2022 Elsevier. **D** The baicalin (BA) encapsulated EV fabricated using sonication and incubation method. Reproduced with permission from [116]. Copyright 2021 Elsevier

The sonication method, in which external ultrasound is applied to induce membrane instability and load drugs into EVs, is also widely used, similar to the method of loading drugs into EVs by applying pressure [110–112]. Resveratrol (Res), a polyphenolic compound that targets NF-κB, exhibits potent anti-inflammatory effects and is utilized in various disorders related to the nervous system. In order to leverage the properties of Res and the targeting capabilities of macrophage-derived EVs, Res was loaded into RAW264.7-derived EVs through a drug loading process using sonication under the conditions of 20% amplitude, 6 cycles of 30 s on/off for 3 min with a cooling period of 2 min between each cycle (Fig. 9A). Subsequently, the membrane stability was restored through incubation at 37 °C for 1 h. After the loading process, Res demonstrated a loading efficiency of approximately 19%. The generated Res loaded EVs (Res EVs) were administered through intranasal delivery in a multiple sclerosis (MS) mouse model. In the MS mouse model, the proinflammatory cytokine (TGF-β, IFN-γ, IL-1β, IL-6, and IL-17) and NF-κB levels were significantly elevated. However, in the Res EV-treated group, a notable reduction in these levels was observed. These findings suggest that the integration treatment with Res and EVs exhibited a substantially enhanced therapeutic effect compared to individual treatment with Res or EVs, demonstrating a synergistic and multifaceted function [113]. Zheng et al.’s study involved the use of Mel, a substance known for its anti-inflammatory properties, in combination with M2 macrophages (M2-EVs) (Fig. 9B). Similarly, another study involved the use of Mel, a substance known for its anti-inflammatory properties, in combination with EVs derived from M2 macrophages (M2-EVs). In this study, M2 macrophages were induced using IL-4, and Mel was loaded into isolated M2-EVs by sonication, achieving an efficiency of approximately 35%. When Mel-Me-EVs were applied to inflamed human periodontal ligament cells (hPDLCs), they demonstrated an effect in alleviating excessive stress of the endoplasmic reticulum stress (ER stress) and improving impaired functions. In a periodontitis-induced rat model, they induced osteogenesis and suppressed osteoclastogenesis [114]. In order to enhance the bioavailability and stability of drugs, there are studies involving the introduction of the plasma protein albumin (Alb) into EVs (Fig. 9C). Alb was loaded into EVs by sonication, with any residual Alb removed via mini-SEC. Subsequently, the same sonication method was utilized to load Cur, resulting in final Cur-Alb loaded EVs (CA-EVs) with loading efficiencies of 38% for Alb and 56% for Cur, respectively. In this study, CA-EVs were formulated into the shape of microneedles (MNs); when applied to an imiquimod-induced skin inflammation model, they resulted in the restoration of inflammatory markers to healthy skin levels, suggesting their potential applicability in inflammatory skin conditions [115]. In a study using the potent radical scavenger and neuroprotective agent baicalin (BA), BA was loaded into EVs derived from RAW264.7 cells through exposure to ultrasonic (Fig. 9D). The loading efficiency of BA was approximately 45%. When BA-loaded EVs (BA-EV) were applied to both the transient middle cerebral artery occlusion/reperfusion (tMCAO) model and pMCAO model, BA-EV exhibited accumulation in ischemic regions in both models. This resulting accumulation of BA-EV in the ischemic areas demonstrated the targeting function of EVs loaded with BA into the brain. Furthermore, given BA’s characteristics, when the effect of BA-EV on ROS, it was observed that in an in vitro model of damage induced by oxygen-glucose deprivation (OGD), the BA-EV group mitigated cell damage and exhibited a protective effect against cell death to a greater extent compared to the BA and EV treatment groups. It was further confirmed in an in vivo tMCAO model, where Nrf2/HO-1 pathway regulation was observed. BA-EV post-treatment resulted in increased expression levels of Nrf2, SOD1, GPx1, HO-1, and NQO-1, indicating an upregulation of the Nrf2/HO-1 pathway, which led to ROS suppression, suggesting its potential to mitigate ischemic brain damage [116].

**Fig. 10:**
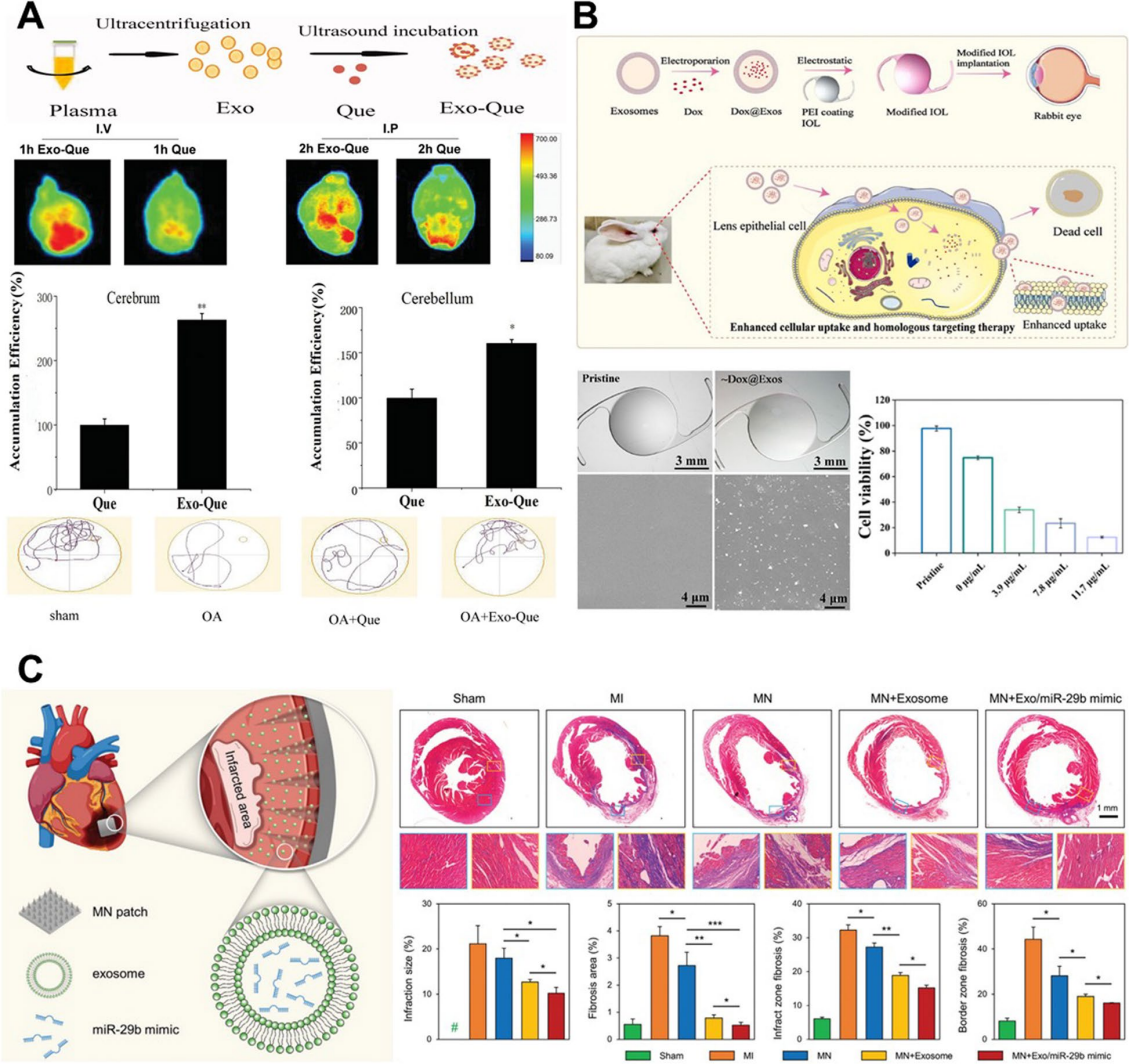
Various applications on regenerative medicine using exogenous engineered EV. **A** The phosphorylated tau-mediated neurofibrillary tangles inhibition in Alzheimer’s disease using quercetin loaded EVs. Reproduced with permission from [117]. Copyright 2020 Informa UK. **B** The therapeutic effect of doxorubicin loaded EVs on posterial capsular opacification. Reproduced with permission from [121]. Copyright 2022 Elsevier. **C** The miR-29b loaded EVs for cardiac fibrosis treatment after myocardial infarction. Reproduced with permission from [123]. Copyright 2023 John Wiley & Sons

Attempts have also been made to deliver quercetin (Que), known for its therapeutic effects on AD, with plasma-derived EVs (Fig. 10A). Que-loaded EVs (Que-EV) were prepared by sonication, and the loading efficiency was approximately 30%. The prepared Que-EV was administered intravenously (i.v) and intraperitoneally (i.p), both demonstrating higher accumulation in the brain compared to free Que., indicating effective targeting. Furthermore, when Que-EV was applied to the mouse model with AD induced by OA, the results from the MWM test indicated that Que-EV had a more effective cognitive improvement compared to free Que. Additionally, Que-EV was shown to inhibit Tau phosphorylation induced by cyclin-dependent kinase 5 (CDK5) more effectively than free Que., thus suppressing the formation of insoluble neurofibrillary tangles, indicating its therapeutic potential in AD [117]. Another method used to load drugs by modulating membrane stability is electroporation [118–120]. This is a method previously utilized for cell transformation or transfection, using a mild electrical stimulus to induce the incorporation of external substances. In a study using lens epithelial cell (LEC)-derived EVs to load DOX, a mixture of DOX and EVs was subjected to electroporation under conditions of 250 V, 350 μF, and 4.5 ms. Subsequently, the membranes were stabilized and restored through a 30 min incubation at 37 °C, followed by a process to remove the free drug, resulting in the production of DOX loaded EVs (DOX-EV, Fig. 10B). In order to suppress the proliferation, migration, and differentiation of residual LECs on the intraocular lens (IOL) surface, a causative factor of posterior capsular opacification (PCO), applied DOX-EVs effectively demonstrated inhibition of cell proliferation and intracellular uptake, showing better results compared to free DOX. To validate these effects in vivo, they fabricated IOLs coated with DOX-EVs and applied them to a rabbit model, effectively demonstrating the suppression of LEC proliferation, and highlighting the suitability of DOX-EVs for addressing PCO [121]. In addition to incorporating various drugs into EVs, there is also diverse research on loading factors such as miRNA into EVs for potential applications. To apply the known inhibitory effects of miR-29 on the TGF-β signaling pathway, miR-29b mimic was electroporated into UCMSC-derived EVs to produce miR-EVs for potential use in cardiac fibrosis induced by myocardial infarction (MI, Fig. 10C) [122]. Treatment of cardiac fibroblasts with TGF-β to induce fibrosis and subsequent treatment with miR-EVs exhibited a significant inhibitory effect on fibrosis. Additionally, a cell migration assay was performed to evaluate its potential to suppress myofibroblast proliferation, revealing suppressed cell migration in the miR-EV group. Based on these findings, miR-EVs were formulated in MN patch form and applied to the infarct zone of the MI mouse model. On the third and seventh day after the application of the MN patch, the expression levels of proinflammatory factors such as IL-1β, IL-6, TNF-α, and iNOS in infarcted hearts were significantly reduced in the miR-EV group. Histological evaluations and the expression of fibrosis-related proteins also showed results closest to the Sham group, indicating the potential for MI treatment [123]. The various exogenous EV engineering methods described above are useful techniques for functionalizing isolated EVs by loading them with a wide range of substances. These methods are applied in various fields and serve as a valuable means of overcoming the limited characteristics of the introduced substances Table 5.

**Table 5:** Exogenous engineering methods for EVs

No.	EV source	Method	Loading material	Ref.
1	HepG2 Cell	Incubation	Bleomycin	[97]
2	MDA-MB-231 cell	Incubation	lactoferrin	[98]
3	Dendritic cell	Incubation	triptolide	[99]
4	ADMSC	Incubation	Ovalbumin	[100]
5	ADMSC	Incubation	Coenzyme Q10	[103]
6	HT92 Cell	Active incubation	Doxorubicin	[104]
7	neutrophil	Extrusion	Doxorubicin	[108]
8	bEnd.3 cell	Sonication	Doxorubicin	[110]
9	RAW 264.7	Sonication	Resveratrol	[113]
10	M2 macrophage	Sonication	Melatonin	[114]
11	M2 macrophage	Sonication	Berberine	[111]
12	J774A.1 cell	Sonication	Curcumin	[115]
13	RAW 264.7	Sonication	Baicalin	[116]
14	HEK293T	Sonication	Erastin/Rose Bengal	[112]
15	Plasma	Sonication	Quercetin	[117]
16	lens epithelial cell	Electroporation	Doxorubicin	[121]
17	Urine	Electroporation	Au NP	[118]
18	Urine	Electroporation	PMA/Fe-HSA@DOX	[119]
19	UCMSC	Electroporation	miR-29b mimic	[123]
20	BMSC	Electroporation	siRNA	[120]
21	BMSC	Electroporation	Rifampicin	[124]
22	BMSC	Freeze-thaw, Incubation, Active incubation, Sonication	bevacizumab	[107]
23	hEnSC	Freeze-thaw, Incubation, Active incubation, Sonication	Atorvastatin	[105]
24	Cabbage	Incubation, lipofectamin	Doxorubicin	[125]
25	UCMSC	Incubation, Freeze-thaw, Extrusion	Paclitaxel	[109]
26	Milk	Incubation, Sonication	Doxorubicin	[126]
27	Panc-1 cell	Incubation, Sonication	Gemcitabine	[127]
28	Milk	Incubation, Active incubation, Sonication	Doxorubicin	[106]

### Hybridization methods for EV engineering

**Fig. 11:**
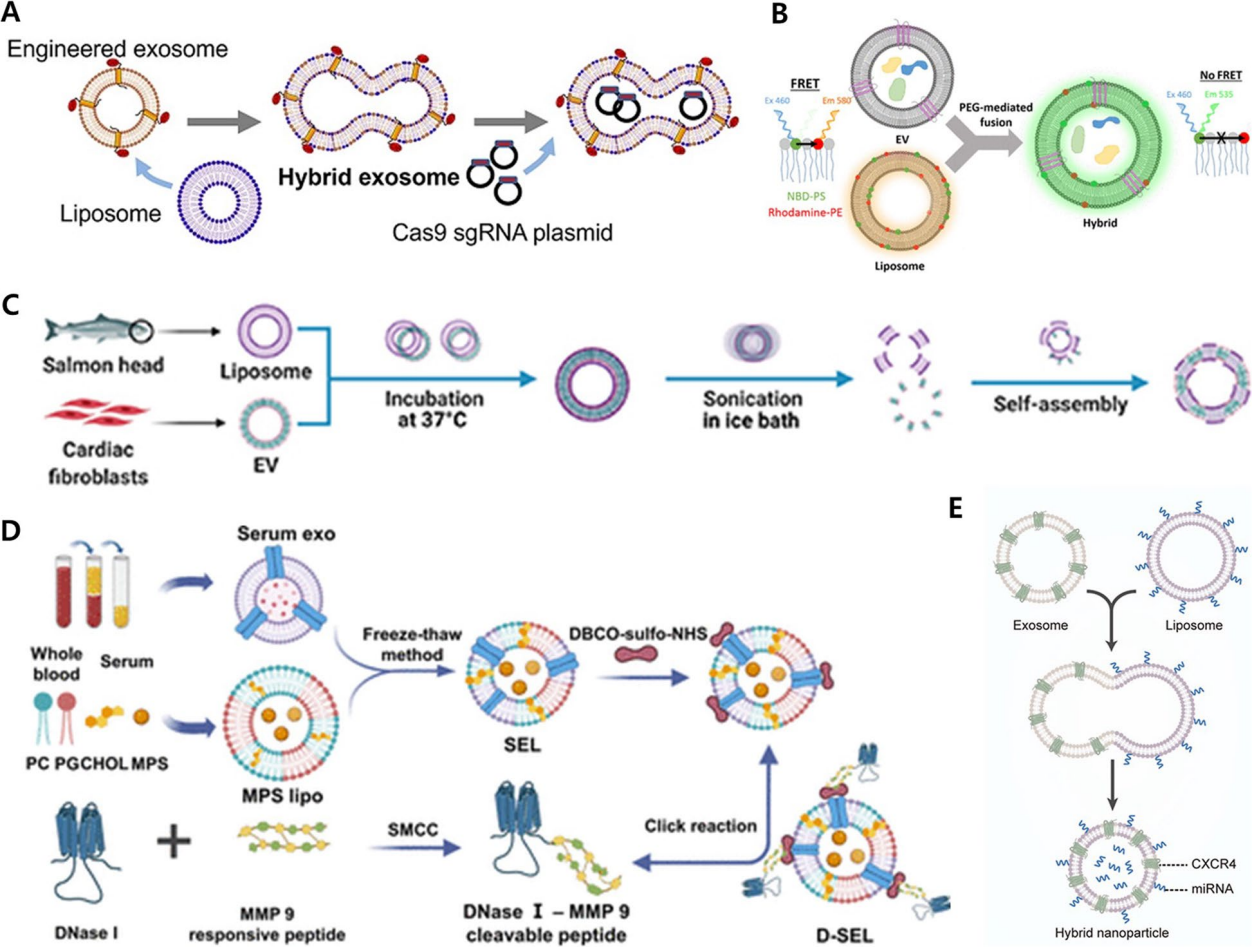
The hybridization methods for EV engineering. **A** The hybridization of EV and liposome using an incubation method. Reproduced with permission from [22]. Copyright 2022 Ivyspring International Publisher. **B** The hybridization of EV and liposome using PEG incubation. Reproduced with permission from [129]. Copyright 2018 American Chemical Society. **C** The hybridization of EV and liposome using extrusion after incubation. Reproduced with permission from [130]. Copyright 2022 IOP Publishing. **D** The hybridization of EV and liposome using freeze and thaw cycle. Reproduced with permission from [131]. Copyright 2023 American Chemical Society. **E** The hybridization of EV and liposome using extrusion. Reproduced with permission from [132]. Copyright 2021 Elsevier

The hybridization method involves reacting isolated EVs with another lipid nanoparticle (LNP) to form a single particle, thereby functionalizing the EVs. Liposomes (Lip) are commonly used in the reaction, and NV, nanoghosts, and EVs are also employed. This method is frequently used to bypass the difficulty of direct engineering of EVs by proteins other than the lipids that make up the EV structure and is often used with established engineering methods. In the hybrid method, the most basic approach is the incubation method [128]. The typical transfection agent, Lipofectamine 2000, is known for components with cationic lipids. In order to load the desired plasmid, Lipofectamine and plasmid were mixed at room temperature for 15 min. Subsequently, the Lipofectamine-plasmid mixture was cultured with EVs at 37 °C for 12 h to produce the hybrid structures. In this study, the used plasmid was the Cas9 sgMMP-13 plasmid, introduced to inhibit the expression of the enzyme MMP13, which is known to exacerbate OA by degrading the extracellular matrix (ECM) in the cartilage region (Fig. 11A). Furthermore, to confer chondrocyte-targeting functionality, DCs were transfected with a CAP-Lamp2b plasmid to express the chondrocyte affinity peptide (CAP). Moreover, transfected DCs were able to successfully isolate EVs that showed CAP on their surface for specific targeting of chondrocytes. Through this process, the hybrid of EVs and Lip was found to be larger compared to EVs and Lip alone, and unlike EVs, which tend to aggregate over time, the hybrid and Lip remained at a consistent size for approximately 24 days. Hybrid formation was validated using a technique known as fluorescence resonance energy transfer. The hybrid produced exhibited efficient cellular uptake, similar to EVs, and this phenomenon was attributed to the presence of CAP on the surfaces of both EVs and hybrids. When applied in vitro, the hybrid demonstrated efficient delivery and led to a reduction in MMP13 expression, resulting in an increase in Col-II expression, a major component of cartilage. Based on these results, the CAP-linked hybrid particles were found to be well-retained in the target joint space when administered via intraarticular (IA) injection in a rat destabilization of the medial meniscus model. Histological analysis revealed the suppression of MMP13, an increase in ACAN and COL II, and the Osteoarthritis Research Society International (OARSI) score returned to a normal level. Therefore, the hybrid particles appear to have a significant therapeutic effect in OA [22].

Similar to the previously mentioned above, the PEG incubation approach is also employed with the introduction of PEG, which is known to promote the fusion of membranes (Fig. 11B) [129, 133]. The PEG incubation method has a reaction time of approximately 2 h, which is considerably shorter compared to the extended 12 h reaction time of the incubation approach, allowing the rapid construction of hybrids. However, the PEG utilized in hybrid formation is an allergenic substance that can potentially induce inflammation and should ultimately be removed, presenting a challenge in the removal process. In addition to the methods mentioned above, techniques such as sonication, freezing and thawing, and extrusion, which are utilized in exogenous engineering approaches for modulating membrane stability, are also employed for the hybrid method. In order to harness the sonication method, Lips were formed using lipids derived from salmon heads, and EVs were isolated from cardiac fibroblasts (Fig. 11C). Separated Lips and EVs were mixed in appropriate proportions and subjected to sonication, resulting in the reconstitution of fragmented particles into hybrids. According to previous studies, when EVs are released from hydrogels, the strength of noncovalent binding decreases by the membrane proteins and the lipid composition of the EVs, and as a result, explosive release is induced [134]. In contrast, Lips exhibit a strong noncovalent interaction with hydrogels, making them advantageous for controlled release. Based on these release characteristics, the hybrid particles produced maintain the diverse functions of EVs while improving the control over the release mediated by Lips [130]. In the research based on freeze and thaw, SEL as the hybrid particle was constructed using liposomes containing methylprednisolone sodium succinate (MPS), a substance that promotes M2 polarization, along with serum-derived EV (Fig. 11D). Subsequently, D-SEL was prepared by attaching DNase I and MMP9 responsive peptide to the surface of the constructed SEL. The particles prepared in this manner were designed for application in inflammatory conditions such as acute lung injury (ALI) or the more severe state of acute respiratory distress syndrome. Given the strong association between inflammation and macrophages and neutrophils in the lungs, the objective was to modulate their roles. It is well known that abnormal formation of neutrophil extracellular traps (NETs), induced by neutrophils, and excessive macrophage activation due to cell death through NETosis are known to be associated with inflammation [135]. Therefore, MPS in D-SEL can be utilized to induce M2 polarization and maintain Tregs’ immunosuppressive function. Additionally, the MMP9 responsive peptide can react with MMP9 expressed in damaged lung cells, causing the release of DNase I, which facilitates the degradation of NETs, thus attenuating inflammation [136, 137]. Indeed, when MPS-loaded D-SEL was applied to the ALI mouse model, a reduction in proinflammatory cytokines such as TNF-α, IL-1β, and IL-6 was observed, while the expression of anti-inflammatory cytokines such as IL-4 and IL-10 increased. As a result of the particle’s NET-degrading capability, gel-forming mucins, MUC5AC, decreased and histological evaluation demonstrated significant amelioration, indicating the therapeutic efficacy against ALI [131]. These results indicate that hybrids produced by the freeze-thaw method can be used in various ways [19, 138–141].

Furthermore, the extrusion method is also one of the widely used techniques [142–144]. This strategy can also be applied to age-related bone loss, which is attributed to the shift from bone formation to fat accumulation in BMSCs due to aging. MiR-188 is known that miR-188 is associated with the promotion of adipogenesis and its inhibition can suppress age-related bone loss in aged mice [145]. Therefore, in this study, Lip containing antagomiR-188, together with EVs derived from NIH-3 T3 cells that overexpress Stromal cell-derived factor 1 (SDF-1), which primarily interacts with the chemokine receptor 4 (CXCR4) known to be expressed in BMSC, was hybridized through extrusion to create particles with multiple functions. Upon IV injection of the constructed particles in vivo, the distribution of the particles in various organs and bones was evaluated 4 h later, revealing the specific localization of hybrid particles in the bone, facilitated by EV-derived CXCR4s. Upon IV injection of the constructed particles in vivo, the particle distribution in various organs and bones was assessed 4 h later, revealing the specific localization of hybrid particles in the bone, facilitated by EV-derived CXCR4s. Moreover, when hybrid particles containing antagomiR-188 were applied to an age-related osteoporosis mouse model, a reduction in cortical bone porosity was observed in micro-CT, suggesting an increase in mechanical strength. Histological analysis revealed that the hybrid particles reduced the number of marrow adipocytes and promoted bone formation, indicating the potential of hybrid particles for the promotion of complex bone formation [132].

**Fig. 12:**
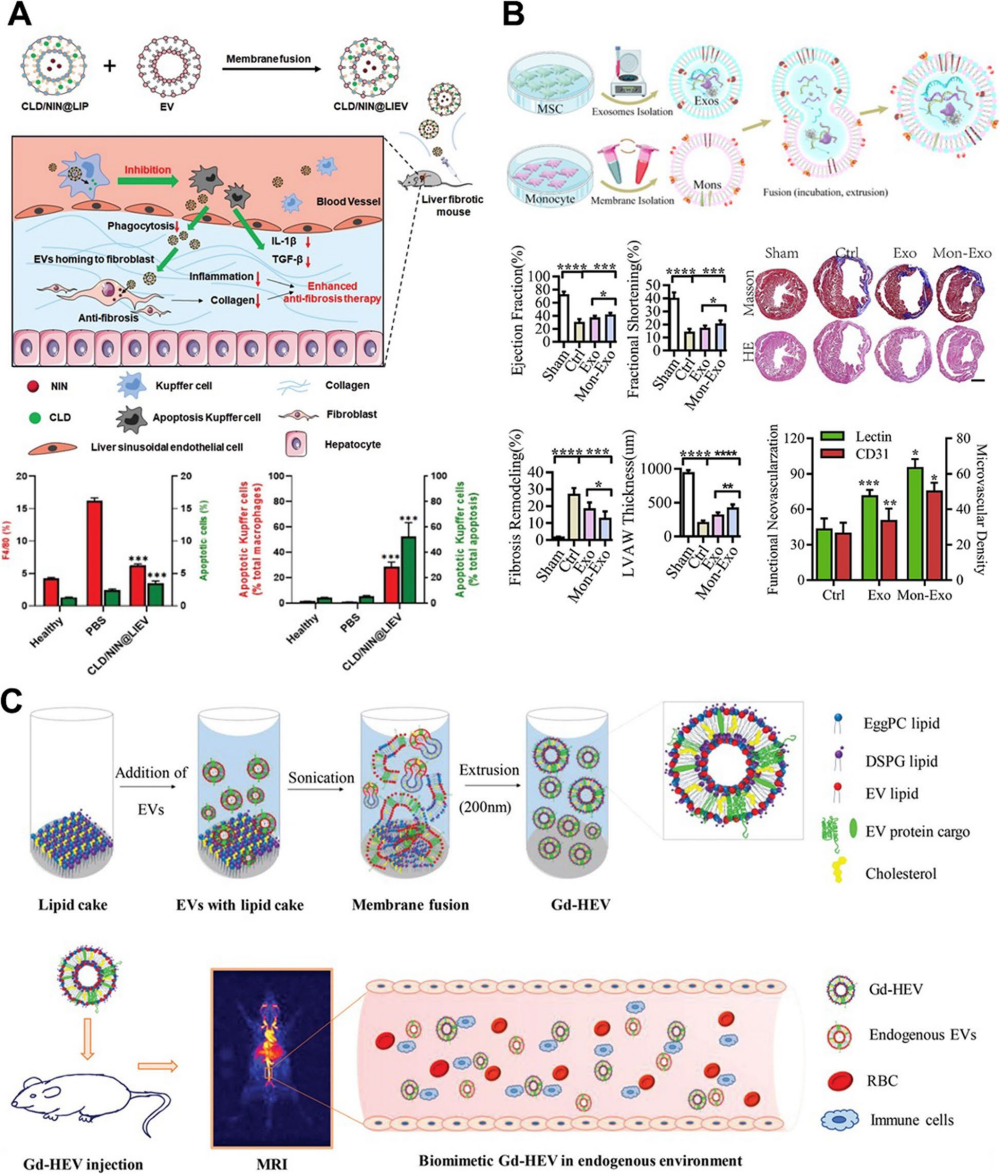
Various applications on regenerative medicine using engineered EVs with hybridization approaches. **A** The hybrid of fibroblast derived EV and clodronate and nintedanib loaded liposome for liver fibrosis therapy. Reproduced with permission from [146]. Copyright 2022 Royal Society of Chemistry. **B** The hybrid of MSC-derived EV and monocyte cell membrane for myocardial ischemia-reperfusion injury. Reproduced with permission from [20]. Copyright 2020 Elsevier. **C** The hybrid of EV and gadolinium infused liposome for advanced magnetic resonance imaging (MRI) contrast agents. Reproduced with permission from [155]. Copyright 2020 Royal Society of Chemistry

This hybrid strategy can also be applied to liver fibrosis. Kupffer cells which exacerbate liver fibrosis with nonspecific phagocytosis can be blocked by nanoparticles as drug carriers. In order to inhibit particle uptake by Kupffer cells, Lip containing the clodronate (CLD) to inhibit macrophage metabolism and intracellular uptake interference, and nintedanib (NIN), to attenuate fibroblast proliferation and activation, and the fibroblast-derived EVs (f-EVs) that have the homing effect, were extruded for the hybrid particles (Fig. 12A). When applied to CCl4-induced liver fibrosis, the produced hybrids led to reductions in collagen deposition, α-SMA-positive fibroblasts, hydroxyproline content, as well as decreased levels of aspartate transaminase and alanine transaminase. This indicates an improvement in liver fibrosis, demonstrating the potential of these hybrids as a therapeutic approach to liver fibrosis [146]. Research has also been conducted using hybrids made by fusion of cell membranes rather than Lips. Cell membranes were isolated from IL-4-induced M2 macrophages and EVs were separated from Annexin A1 (ANXA1) overexpressed Jurkat or EL4 cells, known for their anti-inflammatory properties. The fusion of these cell membrane components and EVs was achieved through extrusion. Jurkat and EL4 cells are T cell lines that can interact with macrophages and induce functional changes. Furthermore, overexpressed ANXA1 can activate macrophage formyl peptide receptor type 2 (FRP2), promoting the expression of IL-10 and consequently inducing M2 polarization. When applied to the mouse model of imiquimod-induced psoriasis-like skin inflammation, the hybrid particles based on these functions significantly alleviated inflammation in the spleen, inhibited macrophage infiltration in skin lesions, and reduced the proinflammatory cytokine levels such as IL-1β, IL-6, and TNF-α, demonstrating their therapeutic potential in treating psoriatic skin inflammation [147]. The incubation method and exogenous engineering methods, including sonication and extrusion, are utilized independently or in combination [148]. In a study involving the application of hybrids for MI/RI, a monocyte membrane was isolated from a monocyte-macrophage cell line of RAW 264.7 cells (Fig. 12B). During the same time, MSC-derived EVs were subjected to a cell membrane to a 15 min incubation at 37 °C and then extruded through a 0.2 μm-sized polycarbonate membrane to create hybrids. The use of monocyte membranes in this context serves to promote recruitment to the ischemic area, as monocytes are a crucial cell type responsible for infiltrating damaged regions to facilitate recovery, which is a characteristic feature of the acute inflammatory response after MI/RI. When hybrid particles composed of MSC-derived EVs showed a regenerative effect, and monocyte membranes were applied to damaged endothelial cells and cardiomyocytes, they exhibited significantly higher cellular uptake efficiency. This shows that the tropism effect mediated by the monocyte membrane of hybrid particles is enhanced under inflammatory conditions in endothelial cells and cardiomyocytes. Based on these results, when hybrid particles were applied to a mouse MI/RI model, the tropism mediated by the monocyte membrane increased the recruitment to the lesion area. This led to the thickness of the recovery of the left ventricular anterior wall, demonstrating the effectiveness of MI/RI [20]. Furthermore, there are studies that combine sonication and extrusion methods to construct hybrid particles [23, 149–153]. This technique has been employed in studies to use these hybrid particles as magnetic resonance imaging (MRI) contrast agents, addressing problems associated with conventional contrast agents such as rapid elimination of MRI contrasts via the renal route, extravasation in the interstitial space, and nephrogenic systemic fibrosis toxicity [154]. This result was achieved by promoting membrane fusion through sonication of the EV macrophage and Gadolinium-infused Lip film and subsequently homogenizing them through extrusion (Fig. 12C). Thus, these hybrids exhibited a significantly higher accumulation within the body over an extended period, facilitated by the characteristics of EV, compared to Gadolinium-infused Lip [155]. Hybrids produced through the exogenous engineering methods mentioned above have shown clear benefits, but they come with a limitation, wherein the engineering process may lead to the loss of internal factors within the EV, preventing them from fully exerting the full range of EV’s effects. In order to overcome these limitations, a recent study comparing several hybridization methods has proposed an ethanol-assisted hybridization method, and the results demonstrated that incubation in a solution containing ethanol efficiently facilitated the fusion of NV and Lip and the successful formation of hybrid particles. This particle formation method is believed to reduce the loss of encapsulated cargo within particles such as EVs or Lips [156]. Furthermore, a more precise method has been proposed to induce one-to-one interactions among particles, facilitating the formation of hybrid particles. This technique involved the attachment of catechols to antibody-targeting markers like CD9. Moreover, due to the characteristics of catechols, particle hybridization could be induced through metal-phenolic coordination when mixed with metal ions such as Fe^3+^. It was validated via two types of EVs, one loaded with Calcein-CO^2+^ and the other with EDTA; the fusion of the two particles facilitated the transfer of CO^2+^ from Calcein to EDTA, utilizing the principle that Calcein fluoresces after this transfer [157]. This precise hybrid technology is expected to preserve the integrity of factors within EVs and holds promise for various applications in different fields. Through various research findings related to hybrid particles, it is anticipated that the limitations of individual utilization of EVs, Lip, NVs, and cell membranes, will facilitate the establishment of a versatile platform technology. Therefore, this technology can leverage the advantages of each particle and find applications in various research fields (Table 6).

**Table 6:** Hybridization for EV engineering

No.	Components for hybridization	Method	Purpose	Ref.
1	EVs / LNP	Incubation	miRNAs encapsulation	[22]
2	EVs / LNP	Incubation	miRNAs encapsulation, Targeting, Circulation	[128]
3	EVs / LNP	PEG incubation	Drug encapsulation, Circulation	[129]
4	EVs / LNP	Sonication	miRNAs encapsulation	[130]
5	EVs / LNP	Freeze and thaw	Drug encapsulation, Targeting, Circulation	[131]
6	EVs / LNP	Freeze and thaw	Drug encapsulation, Circulation	[138]
7	EVs / LNP	Freeze and thaw	Drug encapsulation, Targeting, Circulation	[139]
8	EVs / LNP	Freeze and thaw	Drug encapsulation Targeting Circulation	[141]
9	EVs / LNP	Freeze and thaw	Drug encapsulation Circulation, Targeting	[140]
10	EVs / NVs	Freeze and thaw	Drug encapsulation, Circulation, Targeting	[19]
11	EVs / LNP	Extrusion	miRNAs encapsulation, Targeting	[132]
12	EVs / LNP	Extrusion	Drug encapsulation, Targeting	[146]
13	EVs / Cell membrane	Extrusion	Targeting	[147]
14	EVs / LNP	Extrusion	Drug encapsulation, Circulation, Targeting	[142]
15	EVs / Nanoghost	Extrusion	siRNAs encapsulation, Circulation	[143]
16	EVs / LNP	Extrusion	siRNAs encapsulation, Circulation	[144]
17	EVs / LNP	Extrusion after Incubation	Drug encapsulation, Circulation, Targeting	[148]
18	EVs /Cell membrane	Extrusion after Incubation	Circulation, Targeting	[20]
19	EVs / LNP	Extrusion after Sonication	miRNAs encapsulation, Targeting, Circulation	[149]
20	EVs / LNP	Extrusion after Sonication	Drug encapsulation, Targeting, Circulation	[23]
21	EVs / LNP	Extrusion after Sonication	Drug encapsulation, Targeting, Circulation	[150]
22	EVs / LNP	Extrusion after Sonication	Drug encapsulation, Circulation, Targeting	[151]
23	EVs / LNP	Extrusion after Sonication	Drug encapsulation, Circulation, Targeting	[152]
24	EVs / LNP	Extrusion after Sonication	Drug encapsulation Targeting	[153]
25	EVs / LNP	Extrusion after Sonication	Drug encapsulation, Circulation, Targeting	[155]
26	EVs / NV	Extrusion after Sonication	Drug encapsulation, Targeting	[158]
27	EVs / LNP	EtOH	Drug encapsulation, Targeting	[156]
28	EVs / EVs	Metal-phenolic coordination	Drug encapsulation	[157]

## Conclusion

In this review, we focused on cell culture conditions, isolation methods, engineering methods for extracellular vesicles, and their applications in relation to regenerative medicines. The characteristics of the EVs exhibited significant variations depending on the modulation of the culture conditions, indicating the potential for functional enhancements. Isolation methods have undergone considerable developments, with many techniques currently in use, and improvements are expected in the future. EV engineering can be broadly classified into various methods, including genetic editing, endogenous engineering, exogenous engineering, and hybrid approach. Gene editing is predominantly employed to enhance EV functionality by enabling cells to produce desired factors. Moreover, it can be combined with exogenous engineering and hybrid approaches to further enhance its utility. Additionally, endogenous engineering can be categorized as the inflammation-related factors that induce MSC activation or the other factors. This approach aims to enhance EV functionality based on MSC activation or to induce the incorporation of specific factors into the EVs. As observed in the previously reports, the enhancement of EV functions due to MSC activation largely encompassed regenerative functions such as anti-inflammation, antifibrosis, and anti-apoptosis. Furthermore, exogenous engineering typically involves techniques in which desired factors are loaded into pre-isolated EVs, with many of these methods associated with inducing instability in the EV membrane. The EV membrane instability in these approaches can lead to loss of internal cargo during the engineering process, potentially compromising the full functionality of EVs. Unlike when drug molecules are incorporated into liposomes and used for drug delivery system, stimulations-mediated exogenous engineering methods are risky because EVs contain useful substances both externally and internally. This issue is also observed in hybridization methods that use membrane instability. In order to exclude concerns about side effects due to unexpected changes in cells with genetic modulations or drug treatments, a hybridization approach is promising using clinically applicable liposome components while minimizing the loss of surface or internal materials of naturally secreting EVs. Therefore, several methods have been devised to preserve the internal factors of EVs, which are expected to be highly valuable in the future. Moreover, hybrid approaches serve as a technology that simultaneously complements the drawbacks of individual particles while obtaining their advantages, making them applicable across various approaches. The introduced methods for the engineering of EVs enable functionalization for drug delivery, targeting, circulation, and other applications, facilitating their ease of application in various diseases. This represents an outcome of addressing the limitations of using drugs in isolation or relying solely on the intrinsic functions of EVs. Using these diverse approaches to functionalize EVs, there is a promising outlook for the treatment of numerous diseases beyond kidney regeneration, OA, RA, and nervous system regeneration.

## Data Availability

Not applicable.

## Abbreviations

Ac4ManNAz: N-azidoacetylmannosamine-tetraacetylated
ACAN: Aggrecan
AD: Alzheimer’s disease
ADRs: Adverse drug reactions
AEs: Adverse events
Alb: Albumin
ALI: Acute lung injury
ANXA1: Annexin A1
ATG2B: Autophagy-related protein 2 homolog B
BA: Baicalin
BBB: Blood-brain barrier
CAP: Chondrocyte affinity peptide
CDK5: Cyclin-dependent kinase 5
CKD: Chronic kidney disease
CLD: Clodronate
COL1: Collagen I
COL2: Collagen II
CSMC: Cavernosum smooth muscle cell
Cur: Curcumin
CXCR4: Chemokine receptor 4
CysC: Cystatin C
DBCO-DS: Dibenzocyclooctyne-conjugated dextran sulfate
DC: Dendritic cell
DMOG: Dimethyloxaloylglycine
DOX: Doxorubicin
ECM: Extracellular matrix
Edv: Edaravone
EPO: Erythropoietin
ER stress: Endoplasmic reticulum stress
EtOH: Ethanol
EV: Extracellular vesicle
EXPLORs: Exosomes for protein loading through optically reversible protein–protein interactions.
FBS: Fetal bovine serum
FH: Familial Hypercholesterolemia
FRP2: Formyl peptide receptor type 2
GAG: Glycosaminoglycan
Gem: Gemfibrozil
GFR: Glomerular filtration rate
HDM: House dust mite
Hem: Hemin
hPDLC: Human periodontal ligament cell
i.p: Intraperitoneal
I.v: Intravenously
Que.: Quercetin
IEC: Immune effector cell
IOL: Intraocular lens
ISEV: International Society for Extracellular Vesicles
Ldlr: Low-density lipoprotein receptor
LEC: Lens epithelial cell
Lip: Liposomes
LNP: Lipid nanoparticle
LPS: Lipopolysaccharides
Mel: Melatonin
MI/RI: Myocardial ischemia-reperfusion injury
MI: Myocardial infarction
MJD: Machado-Joseph disease
MLN: Mediastinal lymph node
MNs: Microneedles
MPS: Methylprednisolone sodium succinate
MRI: Magnetic Resonance Imaging
MS: Multiple sclerosis
MSC: Mesenchymal stem cell
MWM: Morris water maze
NEP: Neprilysin
NET: Neutrophil extracellular trap
NIN: Nintedanib
NLRP3: NOD-like receptor protein 3
NV: Nanovesicles
OA: Osteoarthritis
OARSI: Osteoarthritis research society international
OGD: Oxygen-glucose deprivation
PCO: Posterior capsular opacification
PEG: Polyethylene glycol
PKIA: Protein kinase inhibitor α
pMCAO: Permanent middle cerebral artery occlusion
PSD95: Postsynaptic density 95 protein
PTX: Paclitaxel
RA: Rheumatoid arthritis
Res: Resveratrol
RGC: Retinal ganglion cell
RVG: Rabies virus glycoprotein
SAP: Superabsorbent polymer
SCI: Spinal cord injury
SDF-1: Stromal cell-derived factor 1
SEC: Size exclusion chromatography
Slb: Silibinin
SR-A: Scavenger receptor class A
srIκB: Super-repressor IκB
STING: Stimulator of interferon genes
STZ: Streptozotocin
T1D: Type 1 diabetes
TFF: Tangential flow filtration
tMCAO: Transient middle cerebral artery occlusion/reperfusion
TUDCA: Tauroursodeoxycholic acid
Treg: Regulatory T
TSA: Tanshinone IIA
TSG-6: TNF-stimulated gene-6
UC: Ultracentrifugation
UF: Ultrafiltration
XFM: Xeno-free media
